# Age-associated microglial transcriptome leads to diminished immunogenicity and dysregulation of MCT4 and P2RY12/P2RY13 related functions

**DOI:** 10.1038/s41420-025-02295-1

**Published:** 2025-01-19

**Authors:** Martin Škandík, Lara Friess, Guillermo Vázquez-Cabrera, Lily Keane, Kathleen Grabert, Mireia Cruz De los Santos, Mercedes Posada-Pérez, Austeja Baleviciute, Mathilde Cheray, Bertrand Joseph

**Affiliations:** 1 Toxicology Unit, Institute of Environmental Medicine, Karolinska Institutet, Stockholm, Sweden; 2 Department of Oncology-Pathology, Karolinska Institutet, Stockholm, Sweden; 3 Center for Neuromusculoskeletal Restorative Medicine, Hong-Kong, China

**Keywords:** Cellular neuroscience, Microglial cells

## Abstract

The aging process is marked by a time-dependent deterioration in cellular functions, particularly the immune and neural systems. Understanding the phenotype acquisition of microglia, the sentinel immune cells of the brain, is crucial for understanding the nature of age-related neurological diseases. However, the specific phenotype adopted by microglia during aging remains a subject of debate and is contingent on the chosen experimental model. To address these unresolved questions, we employed a novel and highly controlled approach utilizing long-term cultivated BV-2 microglia, exempted from additional external stimuli. Our findings revealed that aged microglial cells, in comparison to their younger counterparts, acquire a distinct gene expression profile, primarily characterized by alterations in microglial immune response. Indeed, pro-inflammatory stimulated aged and young BV-2 microglia exhibited similar transcriptomic profiles, yet the response intensity to the stimulus was markedly muted in the aged microglia. Functional neurotoxic assays confirmed diminished neuronal death in coculture with aged, activated microglia, underscoring a compromised immune response. Furthermore, a subsequent comparative analysis of aged BV-2 microglia with established transcriptomic microglial datasets from aged mice and humans identified 13 overlapping genes, laying the foundation for identifying core microglial aging signature. Particularly noteworthy were *SLC16A3* and *P2RY13*, which consistently exhibited upregulation and downregulation, respectively, across all datasets. Additionally, four other genes—*CAPG*, *LGALS3BP*, *NRIP1*, and *P2RY12*—were found to share regulatory patterns in response to both aging and extrinsic activation. An in-depth investigation focused on *SLC16A3*, encoding the high-affinity lactate transporter MCT4, revealed disruptions in extracellular acidification rate and lactate concentration with age. Microglial purine sensing and motility capacities, regulated by P2RY12/P2RY13, displayed age-related alterations. Remarkably, protein analysis in human brain tissue validated the observed upregulation of MCT4 and downregulation of P2RY12 in aged microglia. In conclusion, our study unveils a distinct phenotype in aged microglia characterized by compromised immune responsiveness. Through the integration of in vitro cultured BV-2 microglia with primary microglia datasets, we identify critical molecular determinants of microglial cellular aging confirmed in human-aged brain tissue. This comprehensive approach offers potential insights for understanding and potentially reprogramming aged microglia, with implications for combating age-related neurological disorders.

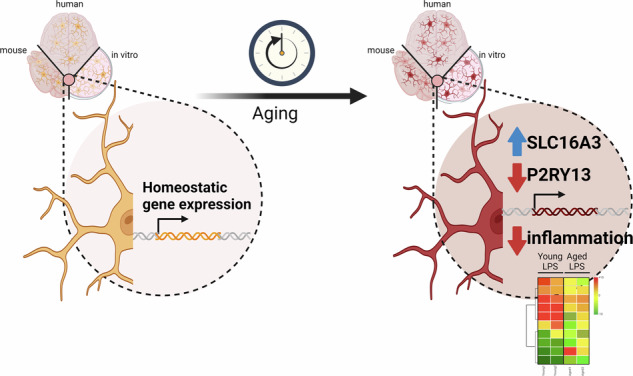

## Introduction

Aging leads to the dysregulation of biological systems, including the nervous system, compromising homoeostasis. Increased oxidative stress and inflammation in the aging nervous system predispose individuals to age-related brain disorders [1, 2]. In fact, aging is a primary risk factor for a spectrum of neurological conditions, including neurodegenerative diseases, cerebrovascular diseases, and brain neoplasms [3–5]. These age-associated brain disorders share a microglial component, wherein microglia, the resident myeloid immune cells of the central nervous system, acquire distinct reactive states with distinct gene expression profiles contributing to pathophysiology [6, 7]. Microglia exhibit transcriptomic and cellular plasticity which are essential for their diverse functions in brain development and the maintenance of a healthy homoeostatic brain [8, 9]. As individuals age, microglia acquire transcriptomic alterations that divert them from their sentinel functions, increasing brain susceptibility to neurological diseases [10–13]. In an aged brain, as compared to a younger one, microglia are reported to display a less ramified cellular morphology, altered immune reactivity with the production of distinct cytokines, impaired ability to sustain synaptic activity, and are often described as a hypersensitized or primed phenotype [6, 14, 15]. Therefore, given the relevance of microglia to various age-related brain disorders, deciphering the microglial aging process has drawn attention from several fields of research.

In mammals, microglia constitute a population of exceptionally long-lived cells, renewing in the human cortex at a median rate of 28% per year [16]. In the mouse cortex, microglia also exhibit longevity, with an average lifespan exceeding 15 months [17]. In addition, microglia as tissue resident immune cells, exhibit a remarkable proliferative capacity. It has been shown that even if the minor residual microglial population is present after depletion attempts, these cells can repopulate the entire brain within days demonstrating the robust self-replenishment capacity [13, 18–20]. Thus, microglia fulfil criteria, i.e., long-lived and proliferative cells, to accumulate age-related damage over time and develop a replicative senescent cellular phenotype. Hallmarks of senescent cells include: expression of p53, p21^WAF1/CIP1^, and p16^INK4A^, coupled to the loss of proliferation capacity; senescence-associated β-galactosidase (SA-β-Gal) activity, the ability to produce secreted factors including matrix metalloproteinases, growth factors, chemokines and pro-inflammatory cytokines, collectively termed the senescence-associated secretory phenotype (SASP); mitochondrial dysfunction linked to increased production of reactive oxygen species; and telomere shortening [21–24] A literature survey reveals conflicting reports on senescence-related markers in microglial models. Primary microglia aged in vitro or isolated from pathology-associated brains show markers of senescence, such as decreased proliferation, upregulation of cell cycle regulators, increased SA-β-Gal activity, and telomere alterations. However, those senescence-associated changes are found to be absent in primary microglial cells acutely isolated from the aged brain or in microglia not associated with a brain pathology [25–27]. Another substantial issue in the field is the choice of experimental model. Indeed, numerous studies originate from the use of aged mammals, that have been exposed, beyond the aging process, to additional challenges that can alter the microglial state of activation and biological function. Animal housing conditions, and their associated challenges, are known to affect animal welfare, physiology, and behaviour [15, 28, 29]. Hence, elucidating the contribution of aging, including the identification of transcriptomic drivers, unbiased from external stimuli to the phenotype acquired by microglia in the aged brain remains topic of intense debate [30, 31]. One of the challenges in addressing these questions relies in the absence of an appropriate model to investigate microglial aging per se, rather than studying microglia isolated from aged mice and humans, i.e., primed microglia exposed to additional challenges.

In this study, we introduce a novel in vitro model of microglial aging, achieved through the long-term cultivation of BV-2 microglial cells, initially established from one week-old murine brains [32]. While microglia isolated from aged mice or humans have typically been labelled as primed microglia due to exposure to both cellular aging and environmental challenges, this engineered in vitro model of microglial aging, exempted from an additional extrinsic activation, allows for the specific investigation of cellular aging processes. The transcriptomes of in vitro aged microglial cells, unchallenged or primed with a proinflammatory challenge, compared to the transcriptomes of primary microglia isolated from aged mouse and human brains, revealed drivers of microglial aging, with associated biological functions found to be altered in the in vitro aged microglia, and whose expressions were found to be accordingly dysregulated in brain tissues for aged human individuals. Overcoming aging model limitations and introducing novel approaches is crucial for advancing our understanding of microglial biology in the context of aging and for developing targeted interventions for age-related neurological disorders.

## Results

### In vitro-aged BV-2 microglia exhibit a unique transcriptomic signature associated with an increase in translation and impaired immune responses

**Fig. 1:**
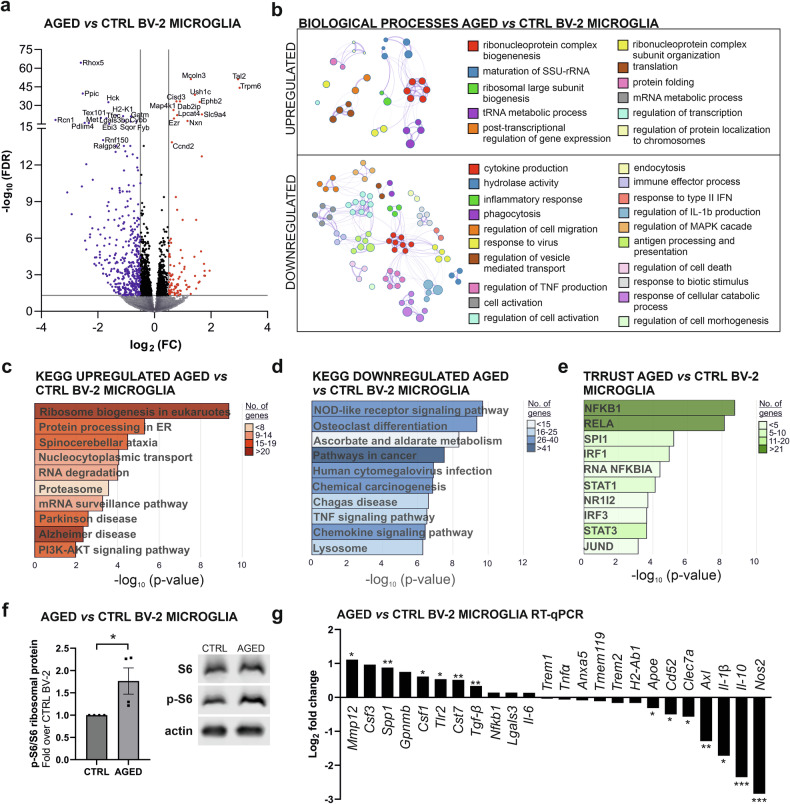
In vitro aged BV-2 microglia exhibit a unique transcriptomic signature associated to an increase in translation and impaired immune responses. **a** Volcano plot illustrating differentially expressed genes (DEGs) based on the log_2_ fold change (FC) related to negative log_10_ false discovery rate (FDR) between aged and young BV-2 microglia. Blue dots represent significantly downregulated genes with log_2_ FC of maximally -0.5, and red dots significantly upregulated genes with log_2_ FC at least 0.5 in aged as compared to young microglial cells. Names of the top genes for up- or downregulated genes ranked according to values of -log_10_ FDR are depicted. **b** Analysis of enriched Gene Ontology (GO) term clusters with FDR < 0.0001 showing nodes grouped by colour in clusters based on their similarity. Lines between nodes represent number of genes overlapping in different nodes. KEGG pathway enrichment analysis that allows identification of significantly affected pathways related to upregulated genes (**c**) or downregulated genes (**d**) between aged BV-2 microglia and controls. **e** TRRUST analysis, which allows the identification of potentially involved transcription factors based on the expression of their target genes in aged BV-2 compared to control cells. **f** Level of phosphorylation of S6 ribosomal protein, part of ribosomal 40S subunit, measured by immunoblot assay in aged and control BV-2 cells. **g** List of genes involved in alternative and classical microglia priming/activation in aging showing FC in aged BV-2 microglia as compared to controls. Data are mean ± SEM from 4 (**f**) and 3–5 (**g**) independent biological replicates. Statistical annotations *p < 0.05; **p < 0.01***p < 0.001; for the indicated comparison.

Which phenotype microglia, long-lived resident immune cells of the brain, acquire upon aging, remains a matter of debate. One major issue resides in the models used to study microglial aging. Indeed, studies have been primarily based on the use of microglia collected from aged mammals, which beyond being exposed to the aging process per se, encounter additional challenges that could alter the state of activation and biological functions of these immune cells [33, 34]. To circumvent this issue, we developed an entirely novel in vitro model for microglial aging based on the long-term cultivation, without any additional challenges, of the juvenile mouse BV-2 microglial cell line established from 1-week-old mixed brain cultures obtained from C57BL/6 mice [32]. Long-term cultivation is a well-established method for inducing replicative senescence and thus age-related cellular phenotype [35]. The mouse BV-2 microglial cells were subjected to extended cultivation exceeding 100 days in culture, corresponding to 130 cumulative population doublings, to generate aged BV-2 microglia. To evaluate plausible transcriptomic alterations associated to aged BV-2 microglia as compared to their control counterparts, high-throughput RNA sequencing (RNA-Seq) was performed on 5 independent replicates for aged and control BV-2 microglia. Control BV-2 microglia, i.e. young BV-2 microglia, were characterized by passages P5-P10*,* i.e., the number of times the cell culture has been subcultured. Bioinformatic analysis reveals that aged and control BV-2 microglia exhibit different gene expression profiles with 1681 differentially expressed genes (DEGs) between the two groups (*p* values adjusted for false discovery rates, FDR < 0.05) (Supplementary Table 1). A volcano plot illustrates the upregulation of 603 genes and the downregulation of 1078 genes in aged BV-2 microglia when compared to control BV-2 microglia (Fig. 1a). The top 30 upregulated and downregulated DEGs sorted by fold change are presented in Supplementary Fig. 1.

Cytoscape network analysis of gene ontology (GO) terms employing an enrichment map for DEGs between aged and control BV-2 microglia, unveils that up-regulated genes in aged BV-2 microglia were associated with GO enrichment for biological processes such as RNA processing and engagement of the translation initiation machinery, particularly in relation to ribonucleoprotein complex hits. The analysis of down-regulated genes in aged BV-2 microglia as compared to their control counterpart implies a dysregulation of the microglial immune response manifested by alterations in ‘cytokine production’, ‘inflammatory response’, ‘regulation of cell migration’, ‘phagocytosis’, and ‘cell activation’ GO terms (Fig. 1b). All corresponding DEGs for the presented clusters can be found in the Supplementary Table 1. Hence, these genome-wide transcriptomic data infer that aged microglia could exhibit a reduced ability to produce an immune response, despite a probable increase in protein synthesis capability.

KEGG (Kyoto Encyclopaedia of Genes and Genomes) PATHWAY analysis that looks at DEGs enriched in selected signalling pathways covering a range of biological processes, further supports the concept of reduced immunogenicity for aged BV-2 microglia. Indeed, KEGG PATHWAY analysis that compared the DEGs between long-term cultivated BV-2 microglia and control cells at a low passage reveals enrichment for pathways associated with protein production but a decline for pathways linked to immune responses (Fig. 1c and d). Pathways associated with Parkinson’s or Alzheimer’s diseases, the two most common age-related neurodegenerative diseases, are also found upregulated in aged BV-2 microglia (Fig. 1c). In contrast, signalling pathways involved in recognition of non-self-components, namely nucleotide-binding oligomerization domain (NOD)-like as well as pathways for chemokine and tumour necrosis factor (TNF) signalling, are found to be downregulated (Fig. 1d; Supplementary Table 1), highlighting a unique age-related immunocompromised microglial phenotype. Transcription factors and their target genes are key players in the transcriptional regulation and acquisition of transcriptional programs controlling cell phenotypes. Therefore, elucidating transcription factor alterations in aged BV-2 microglia could provide information about the regulatory mechanisms behind their cell phenotype. For this purpose, we took advantage of the TRRUST (transcription factor-target interaction Transcriptional Regulatory Relationships Unravelled by Sentence-based Text mining) database, which allows for the identification of transcription factors potentially involved in cellular responses based on their association with target genes. TRRUST analysis of all DEGs between aged and control BV-2 microglia, independently of their regulation, shows significant differences in the implication for the transcription factors NFKB1, RELA and SPI1, which represented the top 3 entries based on significancy and number of included genes (Fig. 1e; Supplementary Table 1). Worth a note, all three mentioned transcription factors play a crucial role in the regulation of inflammation and cell death.

In line with the genome-wide transcriptomic data suggesting an enhanced translation capacity in aged microglial cells, immunoblot analysis of phosphorylated ribosomal protein S6, part of ribosomal 40S subunit, a downstream of mammalian target of rapamycin complex 1 (mTORC1) [36], indicates increased translation in aged BV-2 microglia, as compared to control BV-2 microglia (Fig. 1f). The transcriptomic data generated by high-throughput RNA-seq also indicates that aged BV-2 microglia exhibit a reduced immune reactive state, as compared to their control counterpart. A reverse transcription quantitative PCR (RT-qPCR) analysis of a panel of murine genes reported to be involved in alternative and classical microglia immune response upon aging [37, 38] confirms the altered transcriptomic immune-related response between aged and control BV-2 microglia. Significant changes are observed in 13 of 24 genes of interest, with *Mmp12, Spp1, Csf1, Tlr2, Cst7* and *Tgfb* showing significant higher expression in aged BV-2 microglia as compared to control BV-2 microglia, while *Apoe, Cd52, Clec7a, Axl, Il1b, Il10, Nos2* show significant downregulation (Fig. 1g).

These genome-wide transcriptomic data provide compelling evidence that aged microglia exhibit a distinct transcriptional profile, indicative of a reduced immune reactive state.

### Long-term cultivated BV-2 microglia lack the acquisition of classical senescent markers

BV2 microglia cells, similar to other immortalized microglia cell lines, can be induced to undergo senescence upon various challenges, including DNA damage, and repeated lipopolysaccharide (LPS) stimulation [31, 39–41]. Surprisingly, we discovered that even after more than 100 DIC, corresponding to 130 cumulative population doublings, BV-2 microglia kept proliferating with a linear increase of cumulative doubling time, and did not reach a plateau phase (Supplementary Fig. 2a). In agreement with this observation, no major differences were observed in cell cycle distribution between aged BV-2 microglia and control BV-2 microglia, when the cellular DNA content was analysed by flow cytometric analysis (Supplementary Fig. 2b, c). Upregulated expression of genes encoding for repressors of cell cycle such as *Cdkn2a* (encoding for cyclin-dependent kinase inhibitor 2A, CDKN2A, also known as p16^INK4A^), *Cdkn1a* (Cyclin-dependent kinase inhibitor 1, CDKN1A, also known as p21^WAF1/CIP1^) and *Tp53* (Cellular tumour antigen p53, p53) are considered as hallmarks of cellular senescence [21]. However, *Cdkn2a* and *Cdkn1a* gene expression were found significantly downregulated in aged BV-2 microglia as compared to young BV-2 microglia, while *Tp53* expression was unaffected (Supplementary Fig. 2d - 2f). A RT-qPCR-based assay revealed longer ends of chromosomes in aged BV-2 microglia when compared to their younger counterpart (Supplementary Fig. 2g). The observed age-dependent increase of telomere length aligns well with the previously noted upregulation of the GO BP term ‘regulation of protein localization to chromosomes’ as depicted in Fig. 1b.

Lysosomal pH-dependent SA-β-Gal together with intracellular acidity measured by Acridine Orange (AO) staining, are biomarkers of cellular senescence [42]. Remarkably, SA-β-Gal activity was not observed in control or aged BV-2 microglia as compared to long-term cultivated WI-38 human lung fibroblast cells that were positive for this marker (Supplementary Fig. 3a). Likewise, in aged BV-2 microglia, AO staining revealed no increase in the red fluorescent signal intensity of AO that may originate from increased acidity of the cellular components, mostly lysosomes (Supplementary Fig. 3b). Mitochondrial mass measured by immunofluorescence upon MitoTracker^®^ staining (Supplementary Fig. 3c) did not show any significant changes for this parameter in the aged BV-2 microglia when compared to control BV-2 microglia. Disruption of the mitochondrial membrane potential during cellular aging, likely due to deficient mitochondrial turnover (i.e., impaired mitophagy) [43], leads to increased production of superoxide anion radicals. The spectrofluorimetric analysis of mitochondrial membrane potential upon staining with Tetramethylrhodamine ethyl ester (TMRE) did not show differences between young and aged BV-2 microglia (Supplementary Fig. 3d). Moreover, aged and control BV-2 microglia exhibit similar mitochondrial superoxide production at both, basal level and upon stimulation with H_2_O_2_ treatment measured by MitoSOX^®^ fluorescent probe (Supplementary Fig. 3e). Hence, these data collectively demonstrate that in agreement with their gene expression profile, aged BV-2 microglia do not exhibit characteristics of aged replicative senescent cells.

We re-analysed previously acquired transcriptomic data obtained from primary microglia isolated from 22-month-old male C57BI/6 J mice using gene expression microarrays (dataset of 6701 age-related significant genes) [44], and from 23-month-old female C57BI/6 J mice using RNA-sequencing (dataset of 887 age-related significant genes) [37]. Additionally, a publicly available meta-analysis of four transcriptomic datasets (813 significant age-related genes) obtained with primary microglia isolated from 24-month-old C57/SJL or DBA/2J mice was included in the comparison of in vitro aged BV-2 microglia with primary microglia isolated from aged mice (i.e., in vivo aging) [45]. A mutual comparison of the three mentioned datasets from primary mouse microglia studies [37, 44, 45] revealed an overlap of 140 genes (Supplementary Fig. 4a). To gain insight into the transcriptomic signature acquired by aged microglia, the above-described microglial transcriptomic data set (for aged BV-2, and primary microglia isolated from aged mouse or aged human) were compared to the gene expression profiles compiled by the Aging Atlas database [46]. To date, the mouse Aging Atlas database comprises 466 aging-related genes and the human one, 500 genes. The comparison to the appropriate (i.e., mouse or human) Aging Atlas database with the transcriptomes of in vitro aged BV-2 (Supplementary Fig. 4b), primary microglia from aged mice datasets [37, 44, 45] (Supplementary Fig. 4c), and primary microglia from aged human brains [11] (Supplementary Fig. 4d) only revealed a rather modest overlap of 68, 17, and 111 aging-related genes, respectively. In addition, we found that the overlapping genes listed in Supplementary Fig. 4b–d do not represent typical gene expression markers for a cellular senescent phenotype or age-associated gene regulation. Instead, the overlapping genes are reported to be implicated in the senescent-associated secretory phenotype (SASP), which is considered part of an inflammatory activation state – immunosenescence; reviewed in [47, 48]. Collectively, these transcriptomic analyses indicate that microglia from aged mouse and human brains, as well as in vitro aged BV-2 microglia, exhibit a gene expression profile related to immunosenescence rather than cellular senescence. Therefore, we decided to further investigate the dysregulated immune reactivity of aged microglia and its role in microglial aging.

### Aged BV-2 microglia exhibit reduced immune responsiveness and neurotoxicity in response to LPS stimulation compared to their younger counterparts

**Fig. 2:**
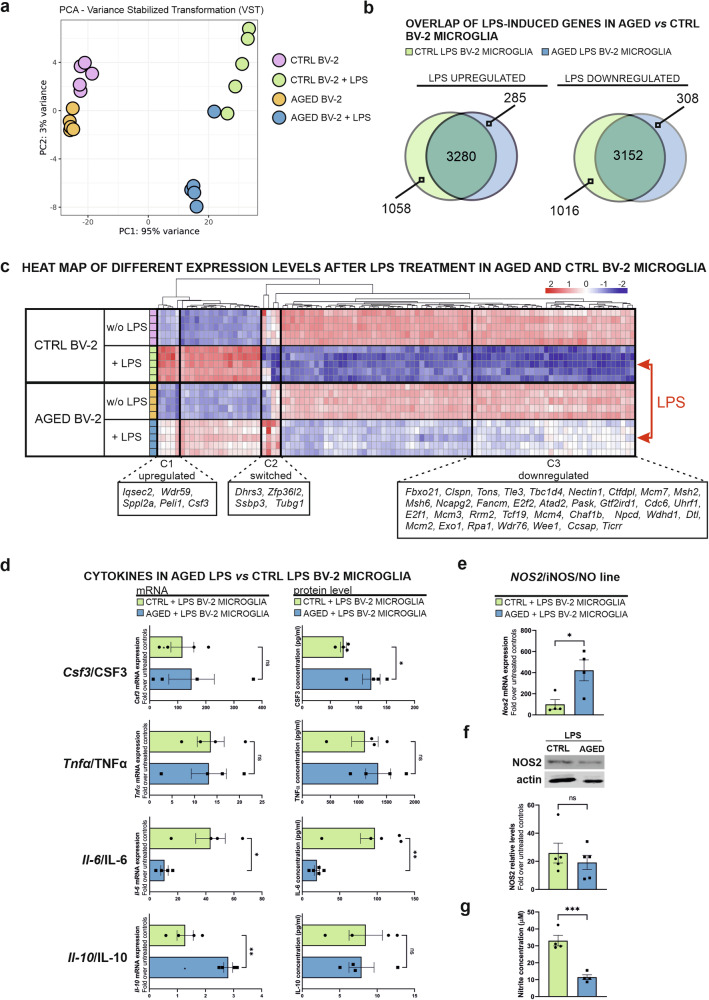
Age-related transcriptomic changes are related to attenuated immune response in LPS-challenged aged BV-2 microglia. **a** PCA plot representing 4 different clusters of samples based on their similarity. **b** Overlap of genes shared by aged and control BV-2 microglia after LPS stimulation. **c** Heat map displaying the top 100 significant DEGs in 5 biological replicates for each experimental condition, untreated or LPS-treated (100 ng/ml for 6 h) aged and control BV-2 microglia showing age-related attenuated expression profile of shared genes. Highlighted clusters display genes that show the highest difference for upregulated (C1) or downregulated (C3) genes in LPS treatment. Cluster C2 displays 4 genes with an opposite regulation upon LPS treatment. **d** mRNA and protein expression levels measured by RT-qPCR and flow cytometry multi-ELISA LEGENDplex, respectively, for *Csf3/*CSF3, *Tnfα*/TNFα, *Il6*/IL-6, and *Il10/*IL-10 in aged versus control BV-2 microglia treated with LPS (100 ng/ml for 6 h). **e** Expression levels of *Nos2* mRNA, (**f**) NOS2 protein, and nitrite (**g**) in aged BV-2 microglia compared to controls upon LPS stimulation are shown. Data are mean ± SEM from 4 (**d**) *Csf3/*CSF3*, Tnfα*/TNFα, *Il6, Il10/*IL-10*;* (e) *Nos2*, and (**g**) NO) or 5 (g) IL-6 and (**f**). Statistical annotations *p < 0.05; **p < 0.01; ***p < 0.001; ns, not significant for the indicated comparison.

Exposing microglia to LPS, a Toll-like receptor 4 (TLR4) ligand derived from gram-negative bacteria, is frequently used to induce a microglial pro-inflammatory reactive state. Upon LPS binding to TLR4, a signalling cascade ensues, leading to the activation of a pro-inflammatory response in microglia, which can result in neurotoxicity and contribute to neurodegeneration [49, 50]. The transcriptomic data described above infer that aged microglia could display a reduced capability to respond to a pro-inflammatory stimulus. To explore this possibility, the gene expression profiles of aged *versus* young BV-2 microglia exposed to the inflammogen LPS for 6 hours were investigated by RNA-seq following an identical procedure and sample size, 5 replicates, as described before (Supplementary Table 2). Principal component analysis (PCA) exposes four distinct clusters representing a unique transcriptome for each experimental condition, i.e., control BV-2 microglia treated or not with LPS, and aged BV-2 microglia exposed or not to LPS (Fig. 2a). The top 30 up- and downregulated DEGs sorted by a fold change are presented in Supplementary Fig. 5. LPS stimulation induces a transcriptomic response in both aged and control BV-2 microglia. In the young control BV-2 microglia, 8506 DEGs (4338 up- and 4168 downregulated) are observed between the LPS-treated and untreated groups. Treatment of aged BV-2 microglia with LPS reveals a lower number of DEGs, 7025 (3565 up- and 3460 downregulated) between the stimulated and unstimulated cells (Supplementary Table 2). Mutual comparison of the LPS-treated aged BV-2 microglia with the LPS-treated control BV-2 microglia shows an overlap for 3280 upregulated DEGs and 3152 downregulated DEGs (Fig. 2b). Hence, the gene expression profiles of LPS-stimulated aged and young BV-2 microglia exhibit significant overlap in terms of DEGs and their regulation, i.e. up- versus downregulation. However, the intensity of the response to the LPS challenge is significantly attenuated in the aged microglia as compared to the control ones (Fig. 2c). Highlighted clusters represent groups of genes with the most significant differences in expression: LPS-upregulated genes (C1) and LPS-downregulated genes (C3). Notably, cluster C2 comprises four genes that exhibit opposite regulation in control and aged BV-2 microglia upon LPS stimulation (Fig. 2c).

**Fig. 3:**
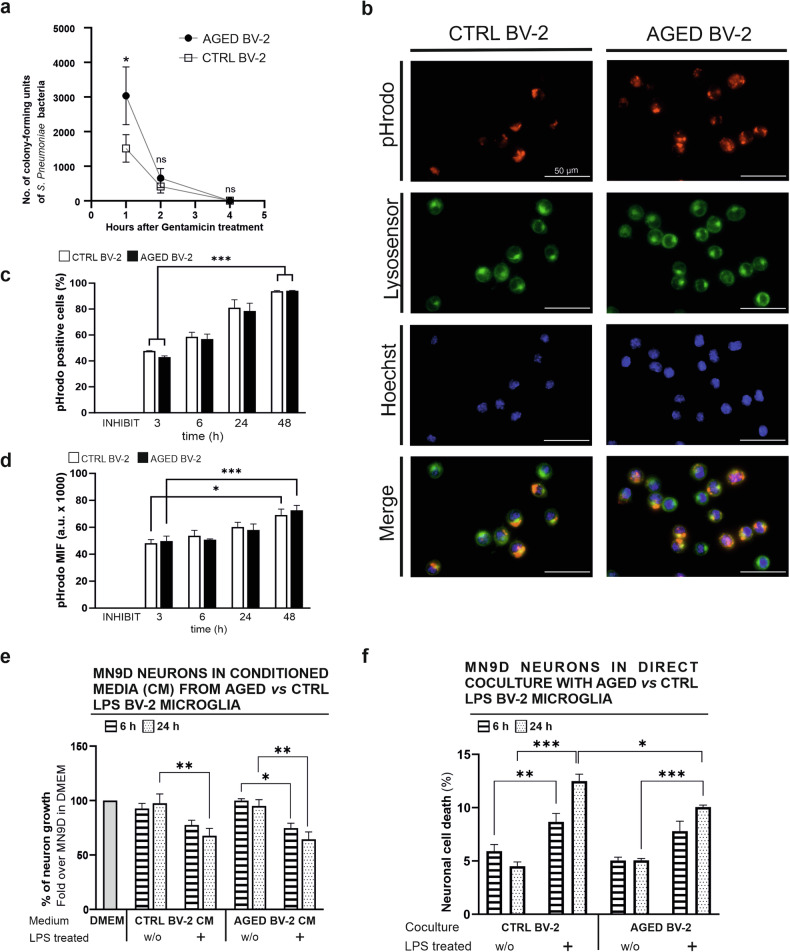
Functional characterisation of aged BV-2 microglia indicates impair bacterial phagocytic capability and reduced neurotoxicity. **a** Internalization and clearance of *S. pneumoniae* bacteria by aged and control BV-2 microglia. **b**–**d** Phagocytic capacity of aged and control BV-2 microglia to internalize phRodo Red-labelled amyloid-β at indicated time points. Scale bar 50 μm. **e** Growth capacity of dopaminergic MN9D neurons cultured for 24 h in conditioned medium from young and aged BV-2 microglia treated with LPS (100 ng/ml, 6 or 24 h) or control condition with PBS/medium only. **f** Neurotoxic effect upon direct co-culture of young or aged BV-2 microglia with dopaminergic MN9D neurons in the absence or presence of LPS (100 ng/ml, 6 or 24 h). Fluorescent-labelled MN9D neurons with abnormal and damaged nuclei were counted and presented as total percentage of cell death. Data are mean ± SEM from 5 (**a**, **e**–24 h time point, **f**), 4 (**e**–6 h time point) or 3 (**b**–**d**) independent biological replicates. Statistical annotations *p < 0.05; **p < 0.01; ***p < 0.001; for the indicated comparison.

Cytoscape network analysis of biological processes related to DEGs in LPS treatment response between aged BV-2 microglia and control BV-2 microglia reveal an upregulation of DNA-related processes like replication, DNA-protein interactions, DNA repair, telomere organisation changes together with cell cycle activation. Downregulated GO terms, similar to the ones uncovered for untreated aged BV-2 microglia in Fig. 1b, are related to microglial immune response, mainly cytokine production (Supplementary Fig. 6, Supplementary Table 2).

The expression levels of several cytokines, including *Csf3/*CSF3 (colony stimulating factor 3, also known as GM-CSF), *Il6/*IL-6 (interleukin 6), *Il10/*IL-10, and *Tnf*/TNF (tumour necrosis factor, also known as TNFα), was investigated by RT-qPCR and the LEGENDplex™ flow cytometric-based assay for secreted protein levels (Fig. 2d). Although *Csf3*, the gene found to be most upregulated in RNA-Seq dataset comparing LPS-related response in aged BV-2 microglia and control BV-2 microglia, does not show different expression by RT-qPCR (Fig. 2d, Supplementary Fig. 5), secreted CSF3 protein levels are significantly elevated in the stimulated aged microglial cells. The induction of *Tnf*/TNFα expression upon LPS treatment in the microglial cells does not significantly differ between the aged and young microglial cells. LPS-treated aged BV-2 microglia exhibit a significant downregulation of *Il6/*IL-6 production and a significant upregulation of *Il10* mRNA expression when compared to the LPS-treated control BV-2 microglia. While IL-10 is considered to be an anti-inflammatory cytokine [51], IL-6 is reported to exert pro-inflammatory effects [52]. Induction of *Nos2*/NOS2 (also known as inducible NOS, iNOS) expression is considered as a feature of microglial cells exposed to an inflammatory stimulus, such as LPS treatment [49]. Whereas *Nos2* mRNA expression levels are significantly increased in LPS-treated aged BV-2 microglia as compared to LPS-treated control BV-2 microglia, in both the RNA-Seq dataset and with the RT-qPCR analysis (Fig. 2e, Supplementary Fig. 5), NOS2 protein expression levels remain comparable between the two experimental conditions (Fig. 2f). Interestingly, a robust reduction in nitrite concentration is observed in the conditioned medium obtained from LPS-treated aged microglial cells in comparison to challenged young microglial cells (Fig. 2g).

Microglia, as professional phagocytes, play a crucial role in clearing pathogens, including bacteria such as *Streptococcus pneumoniae* associated with meningitis [53, 54]. Previously presented data derived from RNA-Seq analysis, followed by GO term analysis, indicate significant alterations in the processes of phagocytosis. Additionally, there is an evident association with biological terms indicative of a reduced response to biotic stimuli originating from viral or bacterial source (Fig. 1b, Supplementary Table 1). Thus, the phagocytic response and clearance of the gram-positive *S. pneumoniae* (strand TIGR4) by aged *versus* control BV-2 microglia were assayed. Aged BV-2 microglia exhibit a higher intracellular bacteria content at 1 hour, which was not sustained at later time points (2 and 4 hour), as compared to control BV-2 microglia (Fig. 3a) suggesting an altered age-associated phagocytosis of bacteria and bacterial clearance capabilities at early time point. Moreover, microglia can be involved in the clearance of misfolded proteins such as amyloid-β peptide (Aβ) aggregates in the context of Alzheimer’s disease (AD) and thus regulate the progression of the disease [50, 55]. Aged and control BV-2 microglia showed similar uptake of fluorescently labelled Aβ (pH-sensitive phRodo Red dye) over time (Fig. 3b, c). Moreover, we did not observe changes in the mean fluorescent intensity of pHrodo Red measured by flow cytometry, which indicates similar acidity of the intracellular compartment [56] (Fig. 3d).

The impact of the secretome from untreated and LPS-treated aged versus control BV-2 microglia on neuronal cells, was evaluated by cultivating dopaminergic MN9D neurons in conditioned medium collected from microglial cell cultures. The conditioned media from LPS-treated aged and control BV-2 microglia significantly inhibit MN9D neuron growth after 24 hours, an effect observed even at 6 hours with conditioned medium from LPS-treated aged BV2 microglia (Fig. 3e). Direct assessment of neurotoxicity, i.e., neuronal cell death, through mutual co-culture of MN9D neurons with aged or control BV-2 microglia followed by LPS treatment reveals significantly lower neurotoxicity with LPS-treated aged BV-2 microglia as compared to their control counterparts at 24 hours under the same conditions (Fig. 3f). The observed reduced production of proinflammatory *Il*6/IL6 (Fig. 2d) together with decreased nitrite levels (Fig. 2g), in LPS-treated aged BV-2 microglia, compared to the LPS-treated control BV-2 microglia aligns with the finding of reduced neurotoxicity by aged microglia (Fig. 3f).

In summary, these data underline age-related transcriptional disruptions in microglial immune responses. Aged BV-2 microglia demonstrate diminished reactivity to a proinflammatory stimulus, show altered mRNA and protein cytokine expression levels, and consequent attenuation of neurotoxicity as compared to their control counterparts after cell-cell interaction-based co-culture.

### Aged BV-2 microglia share transcriptomic signatures with primary microglia from aged mouse and human brains leading to the identification of microglial aging drivers

**Fig. 4:**
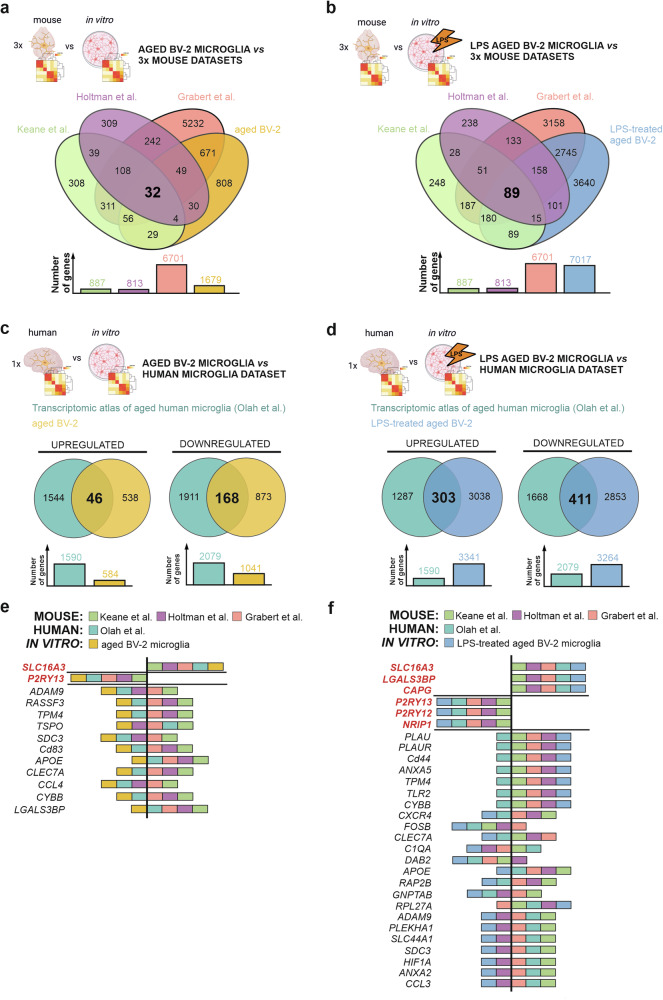
Aged BV-2 microglia show an overlapping gene expression profile with primary microglia from aged mouse and human brains. **a** Venn diagram illustration of the transcriptomic signatures overlaps between unstimulated aged BV-2 microglia and primary microglia isolated from aged mice from three independent microglial aging studies, displaying the number of DEGs found to be differently and commonly regulated between all 4 datasets. The graph under the Venn diagram shows the size of each dataset. **b** Venn diagram representing overlapping and unique DEGs between LPS-treated aged BV-2 microglia and three independent primary mouse microglia aging studies representing common genes between all 4 datasets. Graph under the Venn diagram shows the size of each dataset. **c** Comparison of aged BV-2 microglia and (**d**) LPS-treated aged BV-2 microglia with human primary microglia isolated from elders showing overlapping genes. Graphs under Venn diagram shows the size of each dataset. Graphical representation of shared common genes and its regulation of (**e**) unstimulated aged BV-2 microglia and (**f**) LPS-treated aged BV-2 microglia with primary microglia from mouse and human aging studies.

Aware of the limitations of the in vitro aged BV-2 microglia, and to gain physiological relevance of the findings made with this model, we re-analysed previously acquired transcriptomic data obtained from primary mouse microglia isolated from aged brains [37, 44, 45] (Supplementary Fig. 4a). From the 140 DEGs found in all three aged mouse primary microglia datasets (Supplementary Fig. 4a), 32 genes are found to be differentially expressed in aged BV-2 microglia as compared to their younger counterpart (Fig. 4a). Out of these 32 DEGs, 4 genes (*Cst7*, *Rai4*, *Slc16a3*, and *Spp1*) are found to share common upregulation and 1 gene (*P2ry13*) common downregulation within all compared transcriptomic datasets (Supplementary Fig. 7). Considering that primary microglia isolated from aged mice may not necessarily represent an entirely unchallenged microglia population, but instead a primed one, as reviewed in [6, 7], the transcriptome data sets for aged primary microglia were compared to the transcriptomic data obtained with LPS-treated aged BV-2 microglia. Remarkably, the primary aged microglia share 89 DEGs with the LPS-treated aged BV-2 microglia (Fig. 4b). From this list of DEGs, 39 genes share common upregulation and 3 genes have common downregulation in expression levels across all studied datasets (Supplementary Fig. 8).

The transcriptomic atlas of aged microglia isolated from human brains [11] was used as a template to compare the gene expression profiles of in vitro aged BV-2 microglia and human-aged primary microglia. This analysis shows an overlap of 214 DEGs (46 up- and 168 downregulated gene expression) between the aged mouse BV-2 microglia and the aged human primary microglia (Fig. 4c, Supplementary Table 3). A transcriptomic comparison of LPS-treated aged BV-2 with microglia from aged human brains showed an increased overlap within their gene expression signatures with 714 DEGs (303 up- and 411 downregulated) found in both datasets (Fig. 4d, Supplementary Table 3).

Collectively, these data indicate that primary microglia collected from aged mice and human brain tissues share significantly higher transcriptomic similarity with the LPS-treated, i.e. challenged, aged BV-2 microglia, than with the unstimulated aged BV-2 microglia. In fact, despite well-controlled husbandry conditions, animals kept for 22-24 months should not be considered unchallenged. This concept is certainly also true for primary microglia isolated from an aged human brain that has encountered numerous brain challenges throughout the lifespan of individuals. Thus, primary microglia isolated from both aged mice and humans must be seen as primed cells.

A further meta-analysis of gene expression profiles in the unchallenged in vitro aged BV-2 and the primary microglia from aged mouse and human brains identified 13 shared DEGs across all models. Among those DEGs, two genes exhibited consistent regulation across all datasets – namely, upregulation of *SLC16A3* and downregulation of *P2RY13* gene expressions (Fig. 4e). Additional comparative analyses of the aforementioned primary microglia datasets collected from mouse and human aged brain (which are expected to have encountered additional challenges than just aging), with the transcriptome of in vitro aged microglial cells challenged with LPS were performed. In those conditions, the observed overlap in the DEGs expanded to 29 genes, with six of them exhibiting matching regulation in all datasets (upregulated genes: *SLC16A3, CAPG, LGALS3BP*, and downregulated genes: *P2RY12, P2RY13, NRIP1*) (Fig. 4f).

### Increased *Slc16a3*/MCT4 expression levels in aged microglia is associated with decreased acidification upon LPS treatment

**Fig. 5:**
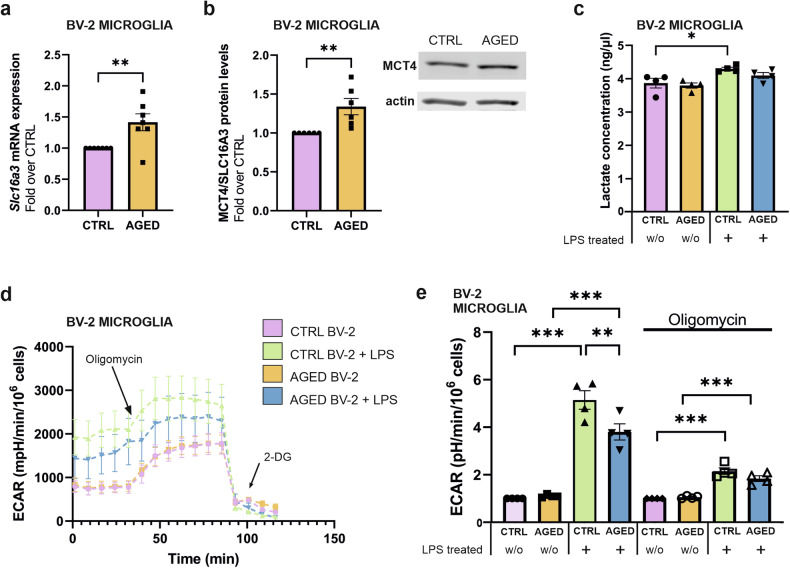
Age-related increase expression of the MCT4 lactate transporter in BV-2 microglia relates to altered extracellular acidification after LPS stimulation. **a** RT-qPCR analysis confirms upregulated *Slc16a3* mRNA expression in aged BV-2 microglia, as compared to control BV2 microglia. **b** Immunoblot analysis demonstrates elevated MCT4 protein levels in aged BV-2 microglia compared to controls. **c** Analysis of lactate concentration in conditioned media originating from aged and control BV-2 microglia with or without LPS treatment (100 ng/ml, 24 h). **d**, **e** Seahorse analysis measurement of extracellular acidification rate (ECAR) in aged BV-2 microglia compared to controls with or without LPS-exposure (100 ng/ml, 24 h). Data are mean ± SEM from 7 (**a**), 6 (**b**) and 4 (**c**, **d**, **e**) independent biological replicates. Statistical annotations *p < 0.05; **p < 0.01; ***p < 0.001; for the indicated comparison.

The *SLC16A3* gene encodes a high-affinity lactate transporter, MCT4 (monocarboylate transporter 4) that hold major role in the export of monocarboxylic acids such as lactate [57]. RT-qPCR and immunoblot analyses were performed to validate the age-related alterations in *Slc16a3*/MCT4 expression levels in BV-2 microglia observed in the RNA-seq datasets. The *Slc16a3* mRNA expression levels exhibit a significant upregulation (Fig. 5a), and MCT4 protein expression levels are found to be significantly increased in aged BV-2 microglia as compared to control BV-2 microglia (Fig. 5b).

Functional verification of an MCT4 alteration, through the direct measurement of lactate concentration in a conditioned medium, did not reveal differences between untreated aged and control BV-2 microglia. However, significantly increased lactate concentration was evident in the conditioned medium originating from LPS-treated control BV-2 cells, an increase not observed in the conditioned medium of LPS-treated aged BV-2 microglia (Fig. 5c). Utilizing the Agilent SeaHorse XF Assay to measure the extracellular acidification rate of live cells, an absence of significant alterations in acidification between aged and control BV-2 microglia was confirmed. Consistent with prior data, aged BV-2 microglia exhibit significantly reduced acidification upon LPS treatment as compared to their young counterparts (Fig. 5d, e).

The observed increase in protein expression (MCT4 encoded by *Scl6a3*) but decrease in the biological function associated to the protein of interest (extracellular acidification) under a proinflammatory challenge, i.e., LPS treatment, is comparable to the effects observed for *Nos2*/NOS_2_ that regulate the production of nitrite by microglia (Fig. 2e, f, g). These data highlight a pattern of increased protein expression, but age-dependent loss of function associated with an immune response.

### Microglial age-related decrease in P2RY12 and P2RY13 in BV-2 microglia indicates impaired migration capabilities and sensitivity

In the context of age-related changes within the microglial transcriptome, the identification of downregulation in the gene expression of *P2RY13*, as well as *P2RY12*, which encode for G protein-coupled membrane purinergic receptors suggest possible age-related changes in microglial nucleotide sensing and migration capabilities. Indeed, the P2RY12 and P2RY13 purinergic receptors can sense extracellular nucleotides ADP and ATP, and thereby direct microglia cell motility [58].

**Fig. 6:**
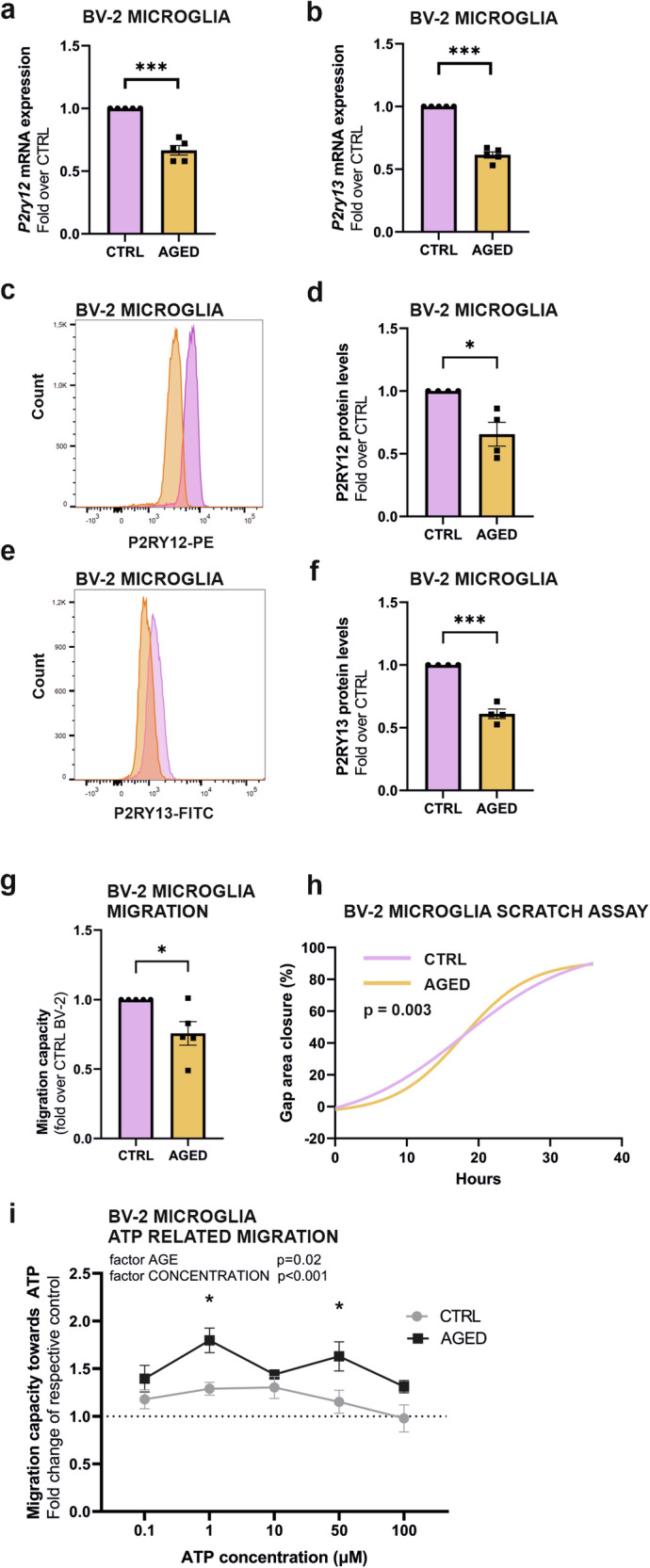
Age-related decrease of P2RY12 and P2RY13 purinergic receptors expression in BV-2 microglia leads to changes in cell motility dynamics. RT-qPCR analysis confirms a significant decrease in *P2ry12* (**a**) and *P2ry13* (**b**) mRNA expression in aged BV-2 microglia compared to controls. Flow cytometry analysis validates the reduction of P2RY12 (**c**, **d**) and P2RY13 (**e**, **f**) protein expression levels in aged BV-2 microglia compared to controls. **g** Transwell migration assay demonstrates age-related changes in microglial migration capacities of aged BV-2 microglia compared to controls. **h** Live cell imaging-based scratch assay reveals alterations in motility dynamics of aged BV-2 microglia compared to controls. **i** Aged and controlled BV-2 microglia exhibit differential motility capacities in response to an ATP gradient, suggesting age-related changes in chemotactic responses mediated by P2RY12 and P2RY13 signalling pathways. Data are mean ± SEM from 5 (**a**, **b**, **g**, **i**), 4 (**d**, **f**), and 3 (**h**) independent biological replicates. Statistical annotations *p < 0.05; **p < 0.01; ***p < 0.001; for the indicated comparison.

The age-related downregulation of *P2ry12* and *P2ry13* mRNA levels was validated by RT-qPCR (Fig. 6a, b) confirming data originating from RNA-Seq. Furthermore, analysis of total protein levels by flow cytometry shows significant downregulation of both, P2RY12 (Fig. 6c, d) and P2RY13 in aged BV-2 microglia compared to control cells (Fig. 6, f). The functional implications for these age-related altered expression levels of these purinergic receptors were tested by two complementary approaches that assess intrinsic and ATP-mediated microglial migration capabilities. First, a transwell migration assay, that used a foetal bovine serum (FBS) gradient as an attractant, demonstrates reduced migration capability of aged BV-2 microglia as compared to control BV-2 microglia after a 24-hour incubation (Fig. 6g). Then, a scratch wounding healing assay coupled to live cell imaging, yielded comparable results, with a detectable difference in the motility rate and subsequent gap closure between aged BV-2 microglia and control BV-2 microglia (Fig. 6h). Migration capabilities in response to varying ATP concentrations, used to stimulate purinergic signalling, were assessed using trans-well system, and reveals a significantly distinct migratory patterns between aged and control BV-2 microglia. Notably, aged BV-2 microglia exhibited a significantly higher rate of migration in response to 1 and 50 μM ATP concentrations (Fig. 6i).

Collectively these data show in microglia, age-related changes in the expression of the *P2ry12* and *P2ry13* genes, which encode for nucleotides-sensing G protein-coupled membrane purinergic receptors involved in the migration capability of these immune cells, a biological function found to be altered in aged microglial cells.

### Aged human brain microglia display an expression profile marked by MCT4 upregulation and P2RY12 downregulation

**Fig. 7:**
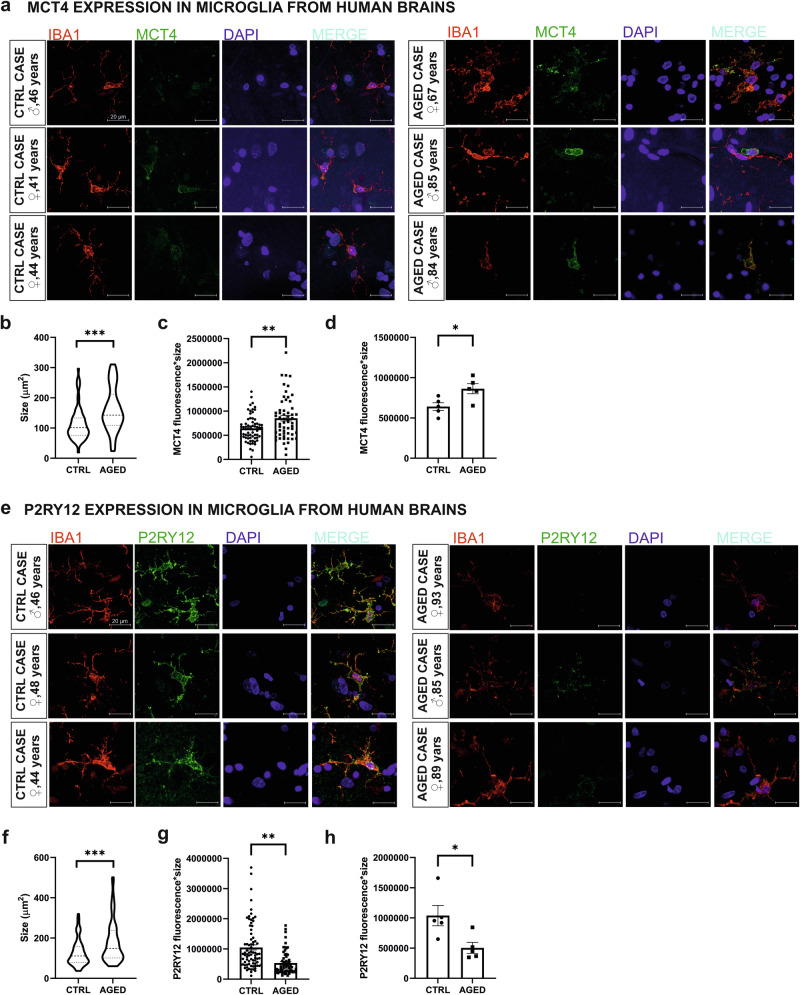
Microglia from elderly human brain tissues display an expression profile marked by MCT4 upregulation and P2RY12 downregulation. **a** Immunofluorescent representative images depict cortical brain tissue from controls and elders labelled with IBA1 (a microglial marker) and MCT4 colocalization. **b** Quantitative analysis reveals alterations in the size of microglia from aged human tissue compared to controls. **c**, **d** Evaluation of single microglia and case-dependent expression patterns demonstrates age-related variations in MCT4 expression, both at the level of individual cells and across different cases. Totally 57 aged microglia and 65 control microglia for MCT4 expression were counted, with a minimum 10 individual microglia per case (**e**) Immunofluorescent staining of control and brain tissue from elders stained for IBA1 and P2RY12. (**f**) Corresponding size analysis on single cell level. **g**, **h** Analysis of P2RY12 expression in individual microglia and across different cases reveals age-dependent variations. Totally 62 aged and 78 control microglia for P2RY12 were counted with a minimum 10 individual microglia per case. Scale bar 20 μm. Data are mean ± SEM from 5 different cases for each age group. Statistical annotations *p < 0.05; **p < 0.01; ***p < 0.001; for the indicated comparison.

The comprehensive meta-analysis of transcriptomic datasets obtained from in vitro aged microglia, unstimulated or treatment with an inflammogen, or obtained from primary microglia isolated from the brain of aged mice or human subjects, allows the identification of candidate genes associated with microglial aging (e.g., SLC16A3, P2RY12, P2RY13). The addition of functional assays associated with the function(s) associated with those microglial candidate genes allows the confirmation of aged-associated microglial alterations (e.g., extracellular acidification, ATP sensing, migration). Collectively, these in vitro, in vivo, and in silico data support an age-dependent upregulation of MCT4 and concomitant downregulation of P2RY12/P2RY13 expression in aged microglia. To gain further human relevance of those findings, the expression level of MCT4, and P2RY12/P2RY13 were investigated in microglia from human postmortem brain cortical tissue samples obtained from the BRAIN UK Biobank. The brain tissue samples from human subjects were divided into two groups: adult brains (median age: 46 years) and brains from elderly individuals (median age: 85 years). MCT4 protein expression in the IBA1-positive microglia is found to be significantly higher in the elderly cases as compared to adult control subjects (Fig. 7a). Complementary to these findings, microglial morphology reveals noticeable morphological changes with increased age. The microglial area demonstrates a significant expansion, accompanied by a reduction in ramification (Fig. 7b and f). To further quantify these observations, analysis of protein expression levels in single microglia cells identified based on IBA1 and MCT4 overlap, encompassing microglial size, confirms the heightened protein levels of MCT4 in tissue from elders compared to control cases (Fig. 7c). Additionally, case-wise comparison underscores the consistent elevation of MCT4 levels in elderly human brains, as depicted in Fig. 7d.

Despite our diligent efforts, the development of a working assay for the detection of P2RY13 protein expression in human brain tissues was proven unsuccessful. Commercially available antibodies, as demonstrated in Supplementary Fig. 9a, failed to yield satisfactory quality and to generate a positive fluorescent signal in our immunofluorescent approach. Our attempts to enhance the signal using a booster were also proved unsuccessful, as depicted in Supplementary Fig. 9b. Considering these challenges, we took the decision to redirect our focus towards an alternative age-related microglial candidate within the same family of proteins, i.e., P2RY12. The immunofluorescence analysis for IBA1 and P2RY12 reveals that P2RY12 protein expression in the IBA1-postive microglia is found to be significantly lower in the elderly cases as compared to adult control subjects (Fig. 7e). Further analysis confirms an age-dependent significant downregulation of P2RY12 expression at the single microglia level (Fig. 7g) and when considered in a subject-wise comparison (Fig. 7h). Representative images originating from all 5 cases for each age group can be found in Supplementary Figs. 10 and 11.

Hence, these investigations confirmed increased MCT4, but decreased P2RY12 protein expression in the cortex of elderly human subjects as compared to adult individuals.

## Discussion

The cellular phenotype and associated functions/dysfunctions acquired by microglia in the aged brain, as well as the cellular biological processes affected and transcriptomic drivers leading to the acquisition of the aged-microglial activation state, are all still a matter of debate in the field. Transcriptomic or proteomic studies of microglia isolated from aged mouse or human brains have shown the existence of distinct gene expression profiles for adult versus young microglia [11, 13, 34, 37, 44, 45, 59].

An experimental approach for cellular aging that is based on upholding cell lines in culture for an extended period of time commonly leads to exhaustion of proliferative capacity by shortening of telomeres, arrest of the cell cycle, changes in morphology, mitochondrial dysfunction and oxidative stress, together with altered lysosomal parameters based on more neutral pH, that are collectively called replicative senescence [24, 60, 61]. A survey of the literature indicates that senescent-related hallmarks are reported for microglia; however, depending on the experimental models used the conclusions drawn vary greatly. Primary microglia aged in vitro and acutely isolated pathology-associated microglia have been reported to present markers of senescence, however, these are not necessarily signs of aging per se. Alzheimer’s disease (AD) is characterised by the deposition of β-amyloid arising during middle-age [62, 63]. Thus, the accumulation of β-amyloid plaque could proceed with the acquisition of the microglial senescent phenotype [27, 64]. In agreement with this hypothesis, microglia exhibiting senescent markers such as SA-β-Gal activity and telomere shortening are primarily found located around the amyloid plaques in the APP/PS1 mouse model of AD [27]. Furthermore, in a cohort of human post-mortem samples from AD patients, a large proportion of amyloid plaque-associated microglia are p16^INK4A^ expressing cells [27, 65]. Likewise, microglia isolated from an amyotrophic lateral sclerosis rat model carrying the SOD^G96A^ mutation show positivity for SA-β-Gal activity, upregulation of p16^INK4A^, and p53 expression [66]. Moreover, foetal human microglia can develop senescent markers such as SA-β-Gal activity, p21 expression, and production of IL-6 and IL-8 as a result of HIV-1 infection [67]. Another discrepancy in the field is based on the choice of experimental microglial model and the use of in vivo/acutely isolated microglia or primary cell cultures that were kept in culture for a few days. While microglia in aged mouse brains in vivo do not present markers of senescence, long-term cultured primary cells show positivity for SA-β-Gal activity, an increase of cell cycle regulators *Cdkn1a/*p21 and *Cdkn2a/*p16, shortening of telomeres, and different regulation of IL-1β, IL-6, and IL- 10 cytokines expression [26]. Thus, the long-term cultivation of BV-2 microglia used in this study represents a unique model of aged microglia that are exempted from any additional stimuli that could prime them and do not acquire classical senescent phenotype, similar to what is reported for primary microglia isolated from the brain of aged mice.

Our genome-wide gene expression analysis revealed a different age-related transcriptomic profile for long-term cultured BV-2 microglia as compared to control, short-term cultivated, BV-2 microglia. Bioinformatic analyses comparing aged, and control BV-2 microglia validated the development of an immune-deficient phenotype in the aged cells. This was evidenced by GO terms reflecting diminished immune responses, particularly those elicited by non-self-components, along with associated signalling pathways. Additional evidence supporting these findings includes differential expression and protein levels of soluble factors (CSF3, IL-6, IL-10) and nitric oxide production. Moreover, chemotactic sensing appears disrupted in aged microglia under basal conditions but exhibits hypersensitivity following ATP stimulation. Thus, aged BV-2 microglia developed a so-called immunosenescent phenotype characterized by an impaired immune reactive state [68]. Microglia express a unique cluster of transcripts encoding proteins for sensing changes in the brain environment, referred to as the microglial sensome [37, 38, 69]. The comparative analysis of the in vivo microglial transcriptomic mouse datasets [37, 44, 45] with the transcriptome for our newly developed in vitro model for aged, unchallenged microglia revealed similar alterations in the expression of microglial sensome genes (including *Clec7a, Csf1, SLC16A3*, and *P2ry13)* [69]. In agreement with those findings, functional assays focused on reactivity to biotic stimulus or microglial neurotoxicity demonstrated an impaired immune response of aged BV-2 microglia. Interestingly, other microglial defensive functions like phagocytosis and lysosomal component, ROS production, and migration are not or minimally affected during aging. Comparable findings were observed in mice acutely isolated from adult and aged cohorts, as well as in vitro studies using primary microglia from neonatal brains. Naturally aged microglia from both, male and females demonstrated heightened phagocytosis of neural debris, but not of beads or *Escherichia coli* bioparticles. Moreover, in vitro aged senescent-like microglia from both sexes exhibited increased internalization of neuronal debris. Following stimulation with interferon-γ (IFN-γ) caused differences in phagocytosis. Natural aging enhanced neuronal debris phagocytosis in both sexes after IFN-γ stimulation, while in vitro aging and IFN-γ treatment resulted in reduced phagocytic capacity of neuronal debris in females [70].

It is important to acknowledge that the aged microglial phenotype observed in humans, including its transcriptomic signature, results from a combination of the aging process, which may vary among individuals, and the reactive states microglia have undergone throughout life in response to diverse brain challenges [33, 71]. Likewise, despite controlled germ-free husbandry conditions, microglia in aged mice have encountered intrinsic and extrinsic stimuli throughout their lifespan that affected their gene expression profile and thereby actual cellular phenotype. Consequently, primary microglia isolated from the aged mammalian central nervous system are therefore to be defined as primed aged cells [6, 37]. Remarkably, transcriptome meta-analysis, revealed that aged BV-2 microglia challenged with a pro-inflammatory stimulus (i.e., LPS exposure), as compared to unchallenged ones, exhibited significantly enhanced gene expression similarities with primary microglia collected from aged mouse or human brain; hence supporting the concept of in vivo aged microglia being primed cells. In contrast, long-term cultivation of BV-2 cells, represents in vitro experimental approach to investigate the cellular aging of microglia per se, without the perturbance of additional challenges that could affect their phenotype. They performed rigorous transcriptome meta-analysis, encompassing microglial datasets derived from aged mouse (6 datasets) and aged human (1 dataset) brains together with transcriptomic datasets obtained from the engineered in vitro aged BV-2 microglial cells exempted from an additional extrinsic activation, or challenged with an inflammogen, allows the identification of genes associated to aged microglia as well as genes to be considered as candidate microglial aging drivers, namely downregulation of *P2RY12* and *P2RY13* and upregulation of *SLC16A3*.

MCT4 encoded by gene *SLC16A3* is involved in lactate secretion. Lactate is continuously produced in the brain and a rise in lactate content is a hallmark of the aged brain [72–74]. Interestingly, *Slc16a3* gene is found to undergo intron retention in astrocytes from aging mouse compared to control animals. The effect of this alternative splicing is not fully understood and requires further evaluation in terms of functionality and MCT4 localization [75]. Maintaining proper lactate levels through the regulation of MCT4 expression is crucial for homoeostasis. For instance, MCT4 expression in astrocytes is essential for preserving long-term memory and spatial learning in the mouse hippocampus [76]. Furthermore, the loss of MCT4 in microglia leads to altered synaptic pruning, increased excitation in hippocampal neurons, and an anxiety-like phenotype in adult mice [57]. Thus, the metabolic crosstalk between astrocytes/microglia and neurons, mediated by lactate, is necessary for proper brain functionality. However, the role of lactate and MCT4 in neurological disorders requires further elucidation [77]. Over recent years, lactate has emerged as metabolite associated with the control of microglial functions including proinflammatory responses, reviewed in [57]. Microglial activation is closely tied to metabolic shifts. When exposed to LPS, microglia display heightened glycolytic activity, akin to the Warburg effect observed in cancer cells [78, 79], while anti-inflammatory IL-4 treatment triggers a reduction in glucose uptake and lactate production [78]. Moreover, inhibition of glucose uptake and glycolysis in pro-inflammatory microglia leads to decreased neuroinflammation [80, 81]. Regulation of inflammation through MCT4 lactate levels was observed in peripheral myeloid cells involved in atherosclerosis, when MCT4 caused metabolic rewiring followed by lactylation of histone H3 on lysine 18 (H3K18la) [82]. Another study describes the role of MCT4-mediated lactylation of histone 4, lysine 12 (H4K12)l in macrophages involved in diabetic cardiomyopathy [83]. Thus, overexpression of MCT4 and increased acidity of the environment based on exported lactate play also a role in the epigenetic regulation of inflammation. The data presented indicates increased mRNA and protein expression of the lactate exporter MCT4 in aged BV-2 microglia, accompanied by similar lactate levels and extracellular acidification rates (ECAR) compared to control microglia. However, disrupted regulation of lactate and extracellular acidification following LPS treatment in aged BV-2 microglia is apparent. The observed decrease in ECAR, indicative of anaerobic glycolysis, may directly correlate with reduced inflammation (as shown by decreased nitrite levels) and LPS-mediated neurotoxicity in aged BV-2 microglia compared to controls. Elevated levels of MCT4 have also been detected in the cerebrospinal fluid of patients with AD at early stages of mild cognitive impairment. Moreover, the overexpression of MCT4 in primary astrocytes has been shown to increase the expression of Aβ42, while concurrently causing a rapid decrease in neuronal growth ability [84].

The P2RY12 and P2RY13 are widely considered homoeostatic microglial markers of non-activated microglial cells [85]. Thus, the observed downregulation of purinergic receptors *P2RY12* and *P2RY13* mRNA and protein expression levels in aging microglia is of particular interest.

Previous reports have shown that microglia located near multiple sclerosis (MS) lesions or amyloid plaques in human brains lose expression of P2RY12, a phenomenon not observed in schizophrenia patients [86]. Additionally, the expression of P2RY12 is restored as the distance from the amyloid plaque increases. Microglia in direct contact with mature amyloid plaques are P2RY12-negative and express proinflammatory mediators. This is followed by microglia with low or negative P2RY12 expression in close proximity to the plaques, while more distanced microglia that fully express P2RY12 help limit the inflamed area [87].

Microglia exhibit multiple motility patterns often triggered by purinergic stimuli. Distinct adenosine diphosphate (ADP)- and adenosine triphosphate (ATP)-activated P2Y purinoceptors control the extension and retraction of microglial cellular processes. Whereas P2RY12, whose encoding gene *P2ry12* was found to be downregulated in aged microglia, responding to low doses of ADP promotes microglial process extension, on the contrary P2RY13, responding to higher purine doses triggers the retraction of these processes [88]. P2RY12 and P2RY13 receptor activation is associated with the expression and release of several proinflammatory cytokines by microglia, e.g. IL-1β, IL-6 and TNF-α in microglia [89, 90].

Hence, the decrease of *P2ry12* and *P2ry13* gene expression and related protein levels observed upon BV-2 microglial aging has impaired related microglial motility capacity. The two identified microglial aging genes, *P2ry13* and *Slc16a3*, are also found to be down- and up-regulated, respectively, in the so-called disease-associated microglia (DAM) that are found in Alzheimer’s disease as well as other neurodegenerative disorders [69, 91, 92]. Worth a note, an upregulation of *Capg, Lgals3pb*, and downregulation of *P2ry12*, defined in this study as aged-microglia genes, are also reported to be part of the DAM signature [91, 92].

We, as authors, are aware of the limitations of long-term cultivated BV-2 microglia cell line as a model system. It was previously reported that BV-2 microglia share the majority of LPS-sensitive genes with primary cells or microglia in vivo, however, the intensity of their response is milder [93, 94]. To overcome this issue, we decided to undertake a metanalysis and compare in vitro aged BV-2 microglia, primary mouse microglia, and human microglia from aged brains to limit the individual effect of BV-2 immortalization, mice housing conditions, the impact of cell culture on the phenotype of primary cells, or external factors involved between human individuals. Furthermore, we limited our investigation to the comparison of the up versus downregulation of expressed genes in the different models used without taking into account differences in the gene expression intensities between those models.

In summary, the development of a novel in vitro model for microglial aging, combined with comparative transcriptomic meta-analysis with primary microglia from aged mouse and human brains, provides insight into the immune deficient phenotype acquired by these cells. These tools allowed us to investigate microglial aging per se, and to identify of potential genetic drivers for this biological process.

## Material and methods

### Cell culture, drug administration and long-term cultivation

Mouse microglial BV-2 cells established from 1-week-old mixed brain cultures obtained from C57BL/6 mice (RRID: CVCL_0182) [32, 93] (gift from Guy Brown, University of Cambridge) were cultured in Dulbeco’s modified Eagle’s medium (DMEM) supplemented with PENSTREP (100 µg/ml penicillin and 100 µg/ml streptomycin) and 10% foetal bovine serum (FBS) (all from Gibco™ ThermoFisher Scientific) at 37 °C in humified 5% CO_2_ atmosphere. Neuronal MN9D cells (RRID: CVCL_M067) [95] (gift from Alfred Heller, University of Chicago) were cultured in DMEM/F12 (Gibco) under the same conditions as above. Cell lines were regularly tested for the absence of Mycoplasma using the LookOut Mycoplasma PCR Detection Kit (Sigma-Aldrich). Cells were treated with 100 ng/ml LPS (from *Escherichia coli*, serotype 026:B6; Sigma-Aldrich) for the indicated time. To develop an in vitro aged microglial model, BV-2 microglia from an initial passage P15 were used. These cells were subsequently cultivated for more than 100 days in culture and were sub-cultivated each time they reached ~ 90% confluency. Thereafter, aged BV-2 microglia were frozen, and aliquots were used for further experiments. BV-2 microglia at low passage, starting from P5, were used as control cells. Both, aged and control BV-2 microglial aliquots were thawed at the same time and subsequently cultivated for max 20 consecutive days.

### RNA isolation and quantitative RT-PCR

Total RNA was isolated using the RNeasy Plus Mini kit (Qiagen) following the manufacturer’s instructions. The yield and quality of the isolated mRNA were analysed using NanoDrop spectrophotometer (ThermoFisher Scientific). The cDNA was synthesized from 1 µg of RNA using Oligo dT, dNTPs, and Superscript IV (Invitrogen) using Bio-Rad T100 PCR Thermal Cycler. RT-qPCR was performed using SYBR Green Master Mix (Applied Biosystems) or SSoAdvanced Universal SYBR Green Supermix (Bio-Rad) and run on StepOnePlus™ Real-Time PCR analyser (Applied Biosystems) or CFX Duet Ral Time PCR System (Bio-Rad), respectively. Results were calculated using Livak’s method and *Actb (β-actin)* expression was used as a housekeeping gene for normalization. The sequences of primers used in this study can be found in Supplementary Table 4. Data are represented as fold-over control BV-2 microglia or untreated control BV-2 microglia in the case of LPS treatment.

### RNA-Seq and computational analysis

RNA was isolated as indicated in the above section with an additional step of DNase treatment. Total RNA samples were subjected to quality control with Agilent Tapestation according to the manufacturer’s instructions. To construct libraries suitable for Illumina sequencing, the Illumina stranded mRNA prep ligation sample preparation protocol was used with a starting concentration of 200 ng total RNA. The protocol includes mRNA isolation, cDNA synthesis, ligation of adapters and amplification of indexed libraries. The yield and quality of the amplified libraries were analysed using Qubit by ThermoFisher Scientific and quality was further checked by using Agilent Tapestation. The indexed cDNA libraries were normalized and combined, and the pools were sequenced on the Illumina Nextseq 2000 for a P2 100 cycle sequencing run, generating paired-end reads. Basecalling and demultiplexing were performed using Illumina bcl2fastq v2.20 software. Reads were aligned to Ensembl GRCm38/mm10 reference genome using STAR v2.6.1 d. Gene counts were estimated using feature Counts software (v1.5.1). Normalization and sample group comparisons of gene counts were performed using R package DESeq2 (v1.28.1). No filtering was performed prior to sample group comparisons, where the default DESeq2 independent filtering was applied.

All differentially expressed genes (DEGs) with a significant adjusted *p*-value (FDR < 0.05), independent of their fold change, were used for further analysis. A volcano plot of up- and down-regulated genes comparing aged versus control BV-2 microglia was created using GraphPad Prism v8. Gene ontology (GO) enrichment maps were created using Cytoscape (v3.7.1, www.cytoscape.org) [96] with the Enrichment Map plugin (https://www.baderlab.org/Software/EnrichmentMap) [97]. GO term gene lists with annotations for biological processes (BP), split by up- and down-regulated in each compared group as described, were downloaded from the bioinformatics database METASCAPE (v3.5, https://metascape.org) [98] and used as input file for the GO network visualization. The individual nodes represent enriched GO terms (FDR < 0.0001). Connecting lines, or edges, represent the degree of overlap between nodes using the default cut-off similarity. The clusters presented were grouped based on their parental BP GO terms to visualize the differences in biological functions between the groups. The PCA graphical representation was created with Rstudio by using ggplot2 package attributes. Venn diagrams to present the overlap of multiple gene sets were performed using the online tool jvenn (http://jvenn.toulouse.inra.fr/app/example.html) [99]. KEGG pathway analysis was performed using EnrichR [100, 101] for up- and downregulated genes. Transcription factor analysis was done using the TRRUST output as part of the EnrichR analysis.

### Immunoblot analysis

Cells were collected directly in 2,5× Laemmli buffer containing Protease Inhibitor Cocktail (Roche) and PhosSTOP (Roche) using a cell scraper. Samples were sonicated (Diagenode, Bioruptor Pico) and boiled, proteins were then separated by SDS–polyacrylamide gel electrophoresis (Mini-Protean tetra Cell system (Bio-Rad)) and blotted onto 0.2 μm pore-size nitrocellulose membranes (Bio-Rad) using the Mini Trans-Blot wet transfer system (Bio-Rad). Membranes were blocked in 5% milk (Semper) in PBS-T [0.1% Tween (Sigma) in PBS (Santa Cruz Biotechnology)]. Primary antibodies anti-S6 Ribosomal Protein (Cell Signalling Technology, 2317S); anti-phospho-S6 Ribosomal Protein (Cell Signalling Technology, 5364 T); anti-NOS2 (Santa Cruz Biotechnology, sc-650); anti-beta-Amyloid, 17-24 (Biolegend, 800701); anti-MCT4 (ThermoFisher Scientific, PA5-99427); anti-Actin (Sigma-Aldrich, A3853) were diluted 1:1000 in 3% BSA in PBS-T and membranes incubated with those antibodies overnight at 4 °C. Membranes were subsequently incubated with RDye® secondary antibodies according to manufacturer’s instructions and visualized by Odyssey CLx infrared imaging system (LI-COR). Obtained fluorescent signals were analysed in Image Studio Lite, version 5.2 (LI-COR) and densitometry was done in ImageJ, version 1.8.0. Levels of all analysed proteins were normalized to Actin. Full immunoblot membranes are presented in Supplementary Fig. 12.

### Cumulative population doublings

After every sub-cultivation event, cells aliquots were stained with trypan blue 0.2% and counted in an automated cell counter Countess II FL (Invitrogen). The cumulative population doublings (CPD) were calculated according to the formula (1):

1 $${CPD}=\,\frac{{days\; in\; cultivation}.\mathrm{log}(2)}{\log \left({final\; concentration}\right)-\log ({initial\; concentration})}$$

### Cell cycle analysis

Cells were collected from the cultivation surface using trypsin, washed with PBS, and fixed in ice-cold 70% ethanol at least overnight at −20 °C. On the day of measurement, ethanol was discarded, and cells were washed with PBS. Subsequently, cells were stained with propidium iodide (PI) solution (10 µg/ml PI (Biolegend), 100 µg/ml RNase A and 0.1% Triton X-100 (Sigma) in PBS) for 15 minutes in 37 °C in the dark. Flow cytometric analyses were carried out on a BD LSR2 cell analyser equipped with the FlowJo v10.8 software (BD Biosciences).

### Telomere length assay

Total DNA was isolated using QIAamp® DNA Mini Kit (Qiagen). Absolute Mouse Telomere Length Quantification qPCR Assay Kit (ScienCell) was used to calculate telomere length based on 2^-∆∆Ct^ method, according to the manufacturer’s instructions. Results are presented as fold-over young cells, and Reference Mouse genomic DNA (4.17 ± 0.1 Mb per diploid cell) supplied by the manufacturer was used for normalization.

### Senescence-associated β-Galactosidase Assay

For SA-β-Gal measurement, the Senescence Detection Kit (Abcam) was used according to the manufacturer’s protocol. In short, cells were cultivated for 10 min in 1X Fixative Solution and afterwards in freshly prepared 1X Staining Solution for overnight incubation at 37 °C without access to CO_2_.

### Acridin orange staining

Acridin Orange (Invitrogen) at a final concentration of 10 µg/ml was added to the culture media and incubated for 20 minutes at 37 °C. A fluorescent signal was detected using an inverted fluorescent microscope Axio Observer 3 (Zeiss) equipped with the Zen blue software (Zeiss). At least 10 pictures were analysed for fluorescent mean for each independent replicate using the ImageJ software (http://imagej.nih.gov/ij).

### Mitochondrial mass

Total amount of mitochondria, i.e. mitochondrial mass, was evaluated upon the addition of 250 nM MitoTracker™ (Invitrogen) fluorescent probe to the culture medium for 30 min at 37 °C. Signal was detected using the inverted fluorescent microscope, described above, and fluorescent mean and area were analysed. At least 8 pictures per independent replicate were used.

### Superoxide (${O}_{2}^{{{\bullet }}-}$) production

Production of mitochondrial superoxide radical was detected using MitoSox™ (Invitrogen) fluorescent probe. 5 µM dye was prepared in HBSS and cells were cultivated for 10 minutes at 37 °C. A fluorescent signal was detected at λ_ex_ 535 nm/λ_em_ 595 nm using a Spark reader (TECAN). Cells treated with 100 µM hydrogen peroxide for 10 min were used as positive control.

### Mitochondrial transmembrane potential

Tetramethylrhodamine, ethyl ester (TMRE, Invitrogen) fluorescent probe at 100 nM final concentration in culture medium for 30 min was used to detect changes in mitochondrial transmembrane potential. The fluorescent signal was measured on a Spark reader (TECAN) at λ_ex_ 535 nm/λ_em_ 595 nm.

### Multiplex cytokine measurement

Conditioned medium was collected from untreated or LPS-treated control and aged BV-2 microglia. Cytokine levels were determined using the LEGENDplex™ mouse inflammatory 13-plex panel (Biolegend, 740446) on a 96-well plate using flow cytometry (BD LSR2 cell analyser) according to the manufacturer’s instructions. The following cytokines were measured: CCL2 (MCP-1), GM-CSF, IFN-β, IFN-γ, IL-1α, IL-1β, IL-6, IL-10, IL-12 (p70), IL17A, IL-23, and TNFα.

### Microglial bacteria phagocytic capacity

*Streptococcus (S.) pneumoniae* TIGR4 strain serotype 4 was provided by Dr Federico Iovino, Karolinska Institutet. Control and aged BV-2 microglia were infected with *S. pneumoniae* (with a multiplicity of infection – MOI 10) for 90 min followed by the addition 200 µg/ml of gentamicin for 1, 2 and 4 h to kill extracellular bacteria. Subsequently, BV-2 microglia were lysed with 1% filtered saponin after indicated time points for 10 min at 37 °C. Then the lysates were diluted 1:10 and 100 µl were plated o/n (37 °C, 5% CO_2_). The next day, colony-forming units were counted.

### Amyloid beta preparation, treatment, and phagocytosis assay

Beta-Amyloid (Aβ 1-42) Aggregation Kit (rPeptide) was used, and aggregation was performed following the manufacturer’s instructions with small adjustments. Final products were evaluated using a thioflavin assay when 5 or 10 µM Aβ monomers were incubated with 10 µM thioflavin overnight. Fluorescent signals at λ_ex_ 430 nm/λ_em_ 485 nm were detected using Spark reader (Supplementary Fig. 13a). Immunoblot analysis was performed to confirm the formation of aggregated products (Supplementary Fig. 13b). Aggregated Aβ was stained with pHrodo™ Red, Succinimidyl Ester (Invitrogen) according to iCell® Microglia Application protocol (FujiFilm Cellular Dynamics) with small adjustments. Young and aged BV-2 cells were treated with stained or unstained 0.5 µM Aβ for indicated time points. Afterwards, cells were collected, washed with PBS, and maintained in HBSS until phagocytic capacity, as well as fluorescent mean, have been analysed using flow cytometry. Representative fluorescent images were performed using Cell Imaging Dishes (Eppendorf) with the same conditions as the phagocytic assay. To visualize lysosomes and nuclei, 1 µM Lysosensor (Sigma) for 30 min and 0.1 mg/ml Hoechst 33342 for 10 min were used.

### Effect of BV-2 microglia conditioned medium on MN9D neuron cell growth

Conditioned medium from control and aged BV-2 microglia, as well as from LPS-treated cells for indicated time points was transferred onto MN9D dopaminergic neurons for 24 h. Afterwards, MN9D cells were collected by trypsinization and stained with 0.2% trypan blue in PBS. Cell numbers were counted using a cell counting chamber and the percentage of the cell population growth was calculated according to the formula:

$$\% \,{of\; growth}=\frac{(x-{C}_{0})}{({C}_{d}-{C}_{0})}.100 \%$$

Where x is the number of cells after treatment, *Cd* is the number of MN9D cells cultivated in fresh DMEM, and *C0* is the number of the MN9D cells at the time of treatment with conditioned medium.

### Effect of BV-2 microglia co-culture on MN9D neuron cell death

MN9D neurons were stained with 2.5 µM CellTracker Green CMFDA (Invitrogen) for 30 min at 37 °C. Then, control and aged BV-2 microglia were seeded over the MN9D cells to generate direct co-culture. When BV-2 microglia were found to be attached to the plastic cell culture ware surface, the co-culture was exposed to LPS treatment for 6 or 24 h. Prior to analysis, cells were stained with 0.1 mg/ml Hoechst 33342 (Invitrogen) for 10 min at 37 °C. At least 6 pictures were taken and a minimum of 100 MN9D cells were counted per independent replicate by inverted fluorescent microscope Axio Observer 3 (ZEISS). The number of dying cells was measured quantitatively by assessing the percentage of cells with fragmented, damaged, or condensed nuclei Supplementary Fig. 14a and b. Representative images of nuclei of dying cells are depicted in Supplementary Fig. 14c.

### Nitrite determination

Levels of nitrite were measured in a conditioned medium from control and aged BV-2 microglia. To reach a detectable concentration of nitrites in the medium, the following experimental conditions were used: 600 000 BV-2 microglia were seeded, and cells were incubated for 17 h with 1 mg/ml LPS. Afterwards, the conditioned medium was collected and frozen at – 80 °C. Analysis was performed following the manufacturer’s protocol for the Griess Reagent Kit for Nitrite Determination (Invitrogen, G-7921). Briefly, the conditioned medium was diluted 1:1 with prepared Griess reagent solution and final absorbance was measured at 548 nm with reference at 690 nm. The final nitrite concentration was measured from the calibration curve of the nitrite standard solution (sodium nitrite) supplied in the kit.

### Lactate concentration measurement

Lactate levels were determined using a conditioned medium from control and aged BV-2 microglia seeded in a concentration reaching confluency. Cells were afterwards exposed to LPS stimulus (100 ng/ml) for 6 h and conditioned media was collected and kept at –80 °C. Analysis was performed according to the manufacturer’s protocol for the Lactate Assay Kit (Sigma-Aldrich, MAK-064). Briefly, conditioned media was mixed with kit reagents and the final colorimetric absorbance of the product was measured at 570 nm with reference at 690 nm and fluorimetric (λex = 535 nm/λem = 595 nm). The final lactate concentration was calculated based on provided standard curve supplied with kit.

### Seahorse

Aged and controlled BV-2 microglia were seeded in a 24-well plate format in media containing 10% FBS. This format was used to avoid bubble formation that could influence measurement. Cells were incubated for 4 h to let them attach to the surface and some wells were subsequently treated with LPS (100 ng/ml) for 24 h. The day after, the medium was changed in DMEM with the same components (with or without LPS) without buffer bicarbonate and 5% FBS and left 1 h in a non-CO_2_ incubator. Sequential injections of oligomycin (5 µM, 8 time points), 2-deoxy-D-glucose (50 mM, 2 time points), and rotenone + antimycin (5 µM, 2 time points) were performed to address mainly the glycolytic metabolism. Several measurements after oligomycin were performed to reach the maximum glycolytic rate. Mixing for 3 min is enough to restore oxygen tension at 155 mmHg and pH at 7.4. Measuring 3 min is appropriate to acquire a proper ECAR measurement.

### Flow cytometry measurement of P2RY12 and P2RY13 expression levels

The concentration of control and aged BV-2 microglia was calculated, and 400,000 cells were used for analysis per experimental condition. Cells were resuspended in flow cytometry buffer (PBS, 0.5% BSA, 5 mM EDTA) and TruStain FcX anti-mouse CD16/32 block (Biolegend, 101320) was used followed by cell permeabilization using 0.1% Triton in PBS for 10 min. Afterwards, cells were incubated with anti-mouse P2RY12-PE (Biolegend, 848003) and P2RY13-FITC (ThermoFisher Scientific, APR-103-F) conjugated antibodies for 20 min. Corresponding rat IgG2b, κ isotpe-PE (Biolegend, 400607) and rabbit IgG-FITC (Invitrogen, 11-4614-80) isotype controls were used. BD FACSCanto II flow cytometry system was used, and data were analysed using FlowJo v10.8.1.

### Transwell migration assay

Cell migration capacity was measured using 8 µm-pore width transparent PET membrane inserts (Falcon) to identify age-related changes in cellular motility towards FBS gradient (5% to 10%) or different ATP concentrations, respectively. The BV-2 microglia of different ages were seeded on top of the insert and let to migrate for 24 hours in an incubator. Subsequently, inserts containing migrated cells were washed in PBS and cells were fixed in 4% PFA. Later, membranes were carefully cut out with a scalpel and mounted with ProLong Gold antifade reagent with DAPI (Life Technologies). Pictures of migrated cell nuclei were taken by inverted fluorescent microscope Axio Observer 3 (ZEISS) and counted using ImageJ.

### Scratch migration assay

Cellular motility was additionally measured by wound healing/scratch assay. The aged and control BV-2 microglia were seeded in 6-well plates in a concentration reaching almost confluency after cultivation for 17 hours. Afterwards, 1000 µl tip was used to make a scratch in the centre of well. Cells were subsequently washed with PBS and kept in FBS-free media. Live cell imaging system (EVOS M7000, ThermoFisher Scientific) was used to take pictures of cells every 2 hours for a 36-hour period. At least 5 pictures in different positions were taken for each experimental group. Images were analysed using ImageJ. Final curves were fitted using Boltzmann transformation using GraphPad.

### Immunofluorescence

Human tissues were first deparaffined by heating slides in an oven at 55 °C for 10 min and then rehydrated using a battery of decreasing concentration of alcohols: 5 min in xylene (2 times), 1:1 xylene:100% ethanol, 100%, 90%, 80%, 70% ethanol and 5 min in distilled water. Antigen retrieval was performed by heat-induced antigen retrieval by immersing the slides in Target Retrieval Solution diluted in distilled water (1:10; Dako, S1699) which were then placed in the 2100 Retriever (Diagnostic technology). Sections were washed in PBS and incubated for 20 min 0.3% PBS-Triton for permeabilization. Sections were washed 2 times in 0.1% PBS-Tween for 5 min followed by 1 h incubation in blocking solution (10% goat serum diluted in 0.1% PBS-Tween) to block unspecific binding. Next, sections were incubated with primary antibodies: rabbit anti-human MCT4 (1:200,ThermoFisher Scientific, 22787-1-AP), rabbit anti-human P2RY13 (1:20, ThermoFisher Scientific, PA5-77675), rabbit anti-human P2RY12 (1:150, Sigma-Aldrich, HPA014518), and mouse anti-human IBA1 (1:500, Synaptic Systems, 234011) diluted in blocking solution overnight at 4 °C. Sections were washed with 0.1% PBS-Tween and followed by 90 min incubation with secondary antibodies goat anti-mouse Alexa Fluor 594 (1:500, ThermoFisher Scientific, A-11005), goat anti-rabbit Alexa Fluor 488 (1:500, ThermoFisher Scientific, A-11008) diluted in blocking solution. Nuclei were counterstained for 10 min with Hoechst 34580 (1:500; Molecular Probes/Fisher, 11584876). Samples were mounted with Fluoromount-G® (Southern Biotechnology/AH Diagnostic, 0100-01) and analysed with Zeiss LSM880 (Karolinska Institutet, Biomedicum Imaging Core).

### Immunofluorescence using booster kit

Tissue rehydration and antigen retrieval were performed as described above. Afterwards, sections were washed in PBS and incubated for 30 min with 99% methanol and 1% H_2_O_2_ to inactivate endogenous peroxidase. After repeated rinses with 0.3% PBS-Triton, human sections were incubated for 1 h in blocking solution (10% goat serum diluted in 0.3% PBST) to block unspecific binding. Next, sections were incubated overnight at 4 °C with corresponding primary antibodies diluted in blocking solution: rabbit anti-human P2RY13 (1:20, ThermoFisher Scientific, 20335-1-AP), rabbit anti-human P2RY13 (1:20, ThermoFisher Scientific, PA5-77675), mouse anti-human IBA1 (1:500, Synaptic Systems, 234011). Sections were washed with PBS and the Alexa Fluor 647 Tyramide SuperBoostTM Kit (ThermoFisher Scientific, B40926) was used to amplify this epitope. Overnight incubation at 4 °C with the biotinylated goat anti-rabbit IgG-HRP secondary antibody solution. Sections were washed with PBS and incubated for 4 min in Tyramide working solution (1x Tyramide stock solution (Alexa Fluor 647), 1x H2O2 solution, 1x Reaction buffer) according to manufacturer’s instructions followed by 7 min incubation with Reaction Stop Reagent Stock solution diluted 1:11 in PBS. Sections were washed in PBS and incubated for 90 min with secondary antibody goat anti-mouse Alexa Fluor 488 (1:200; ThermoFisher Scientific, A-11029) and goat anti-rabbit Alexa Fluor 546 (1:200, ThermoFisher Scientific, A-11010) diluted in blocking solution. Nuclei were counterstained for 10 min with Hoechst 34580 (1:500; Molecular Probes/ThermoFisher Scientific, 11584876). Samples were mounted with Fluoromount-G® (Southern Biotechnology/AH Diagnostic, 0100-01) and analysed with Zeiss LSM880 (Karolinska Institutet, Biomedicum Imaging Core).

### Statistical analyses

Statistical analyses were performed using Prism GraphPad (v8) software and *p* values were calculated between young and aged BV-2 microglia using two-tailed unpaired student’s t-test. Statistical power in experiments composed of different time points was calculated using two-way ANOVA. Statistical power for human brain tissue analysis was calculated using G*Power software (v 3.1.9.7). All experiments were performed between 3 and 6 independent biological replicates. Data are presented as mean ± SEM and considered significant for *p < 0.05, **p < 0.01, ***p < 0.001 between control and aged groups or between LPS-treated groups and untreated controls. All statistical analyses can be found in Supplementary Table 5.

## Supplementary information

Supplementary figures

Original data

Supplementary Table_1

Supplementary Table_2

Supplementary Table_3

Supplementary Table_4

Supplementary Table_5

## Data Availability

The data that supports the findings of this study are available in the supplementary material of this article. The bulk RNA-seq data that support the findings of this study are available at Gene Expression Omnibus under accession number GSE287220. Some of the data that support the findings of this study are openly available in Refs. [11, 37, 44, 45]. Data are available upon reasonable request.
