# Supplementary material for: Age-associated microglial transcriptome leads to diminished immunogenicity and dysregulation of MCT4 and P2RY12/P2RY13 related functions
Source: Cell Death Discov. 2025 Jan 19;11:16. doi: 10.1038/s41420-025-02295-1 (PMC11743796; doi:10.1038/s41420-025-02295-1)
Supplement: Supplementary file 1 — Supplementary figures [file 41420_2025_2295_MOESM1_ESM.pdf]

# Supplementary Figure 1

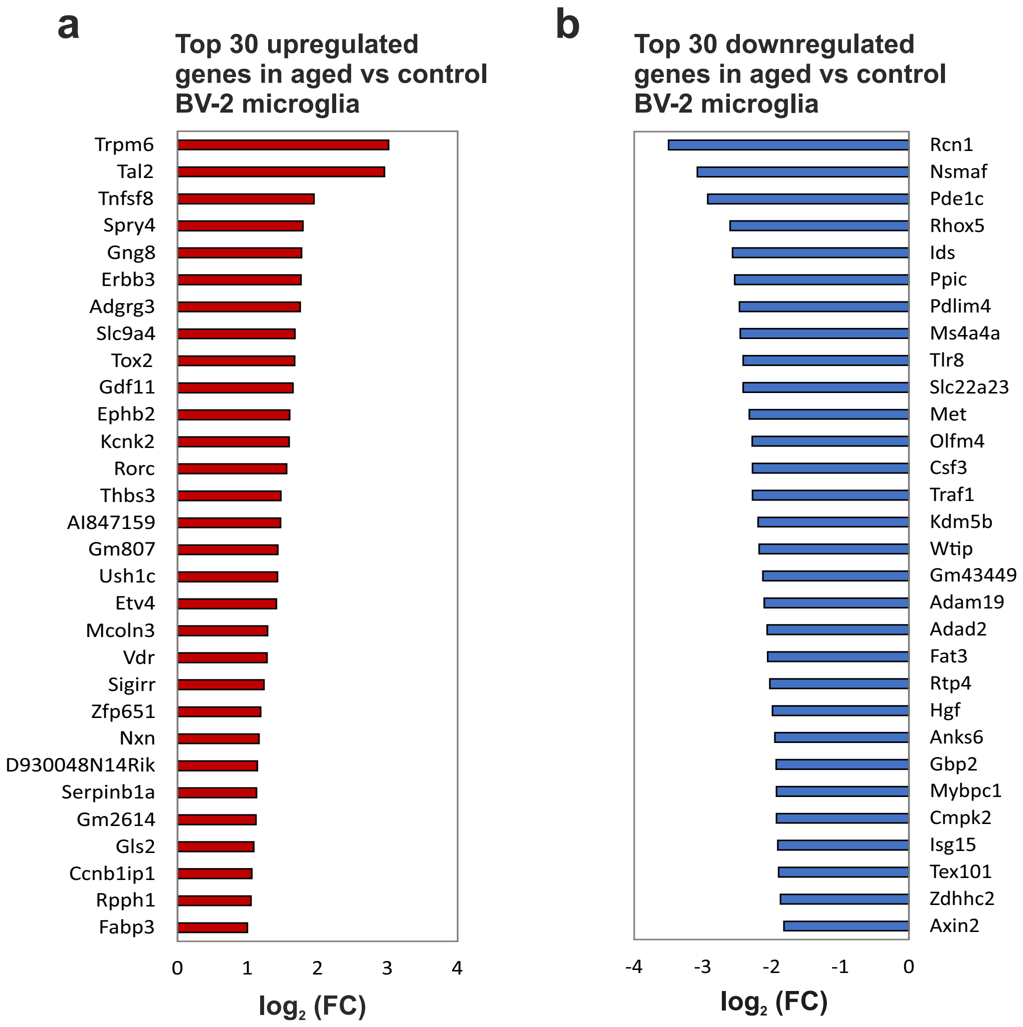


**Supplementary Figure 1** | Graphs display the top 30 upregulated (**a**) and top 30 downregulated (**b**) genes in aged BV-2 microglia compared to control BV-2 microglia.

# Supplementary Figure 2

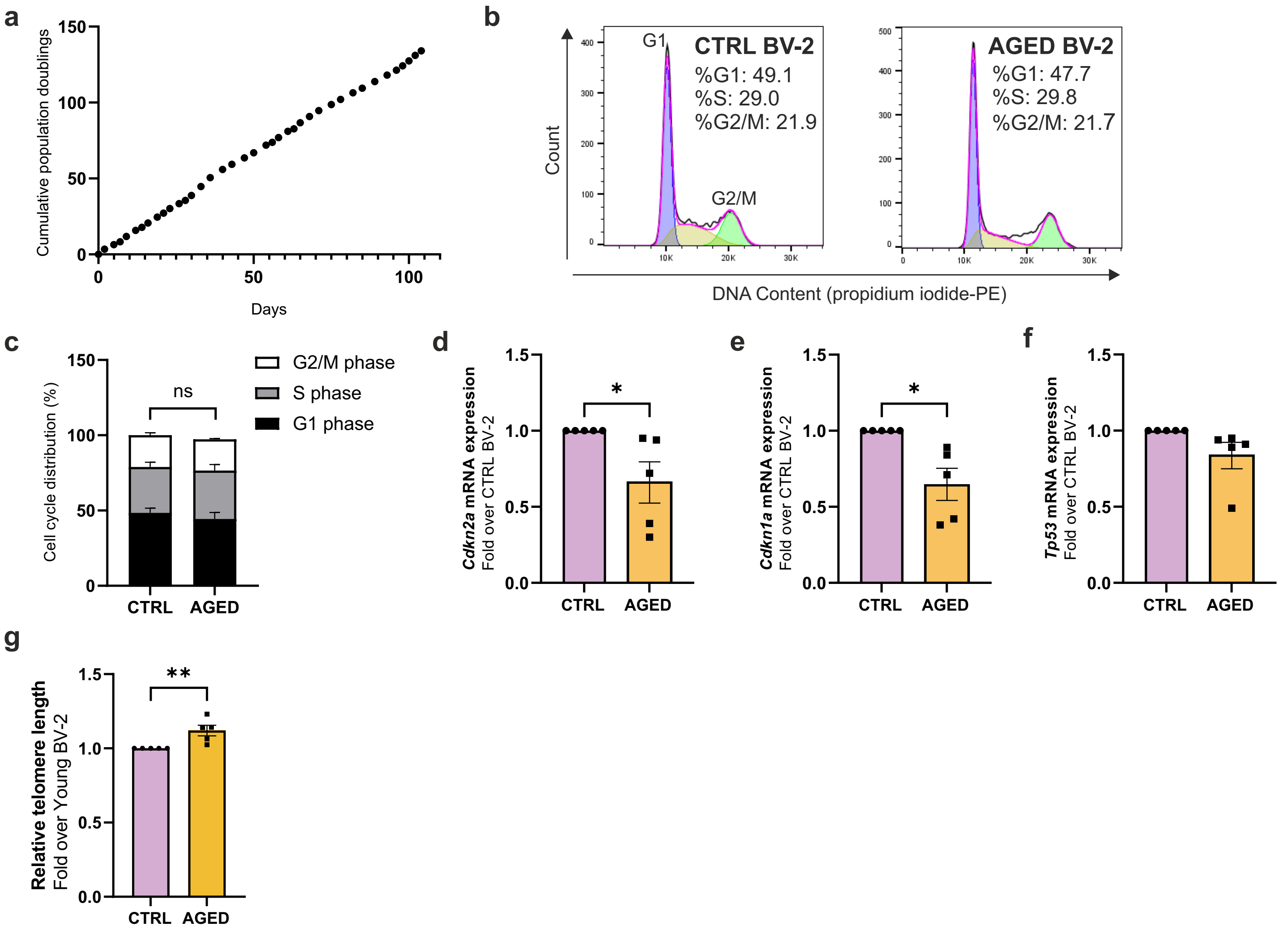


**Supplementary Figure 2 | Long-term cultivated BV-2 microglia hold intact proliferation rate and cell cycle regulation**. (**a**) Effect of a long-term cultivation on cumulative population doublings of BV-2 microglia measured by cell counting. (**b**) Representative histograms and (**c**) quantification of cell cycle analysis by flow cytometry measuring DNA content of aged (>100 DIC) versus control (<14 DIC) BV-2 microglia. (**d, e, f**) Gene expression levels asses by RT-qPCR of cell cycle regulators (**d**) *Cdkn2a*, (**e**) *Cdkn1a*, and (**f**) *Tp53* between aged and control BV-2 microglia. (**g**) Relative telomere length measured by RT-qPCR approach in aged and control BV-2 microglia. Data are mean ± SEM from 5 replicates (**b** to **g**). Statistical annotations *p<0.05; **p < 0.01 for the indicated comparison.

# Supplementary Figure 3

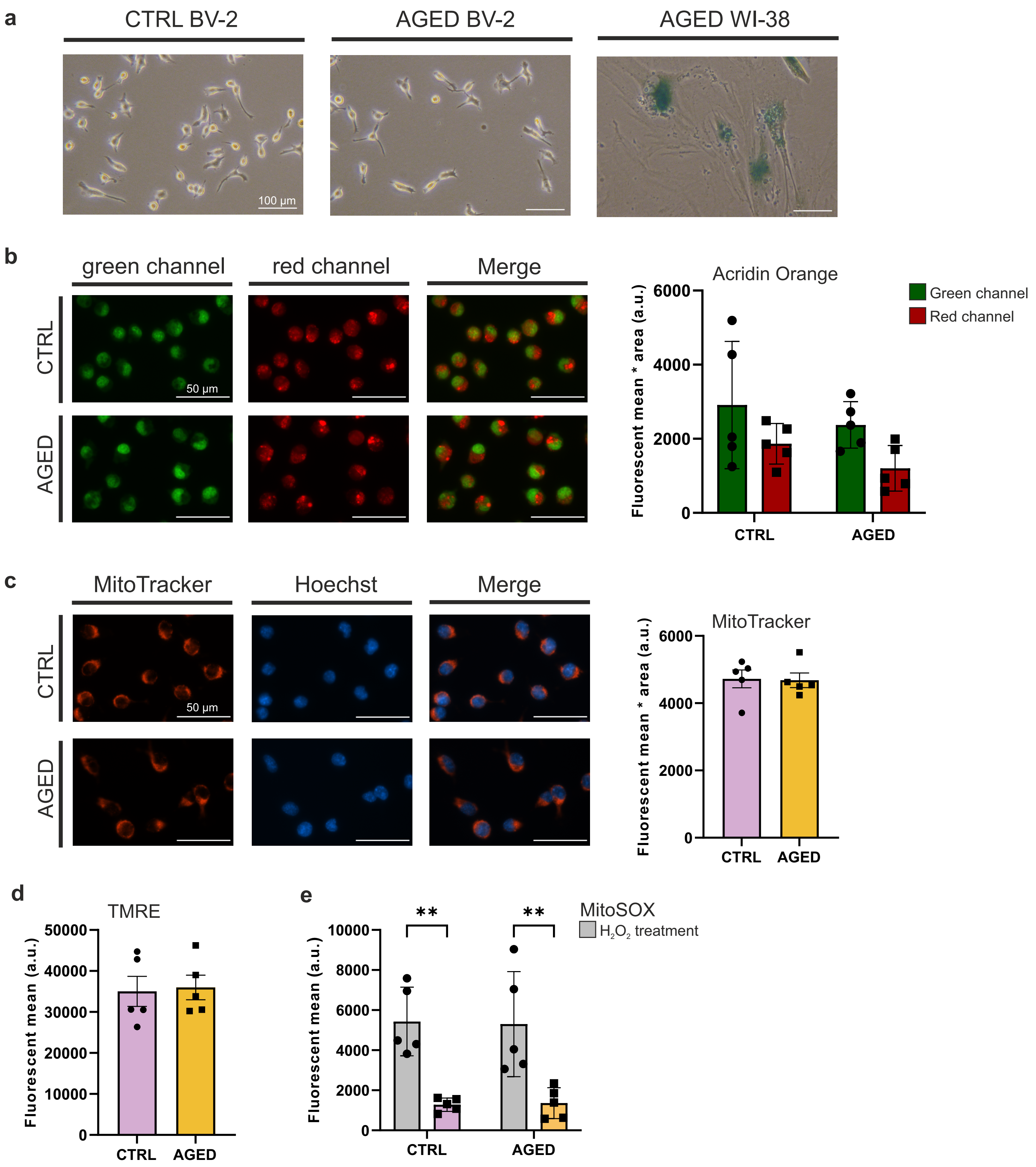


**Supplementary Figure 3 | Absence of senescent-related markers in aged BV-2 microglia.** (**a**) Senescent-associated β-galactosidase (SA-β-gal) activity in aged and control BV-2 microglia. Aged-human lung fibroblasts WI-38 were used as positive control (scale bar 100 µm). (**b**) Representative images of aged and control BV-2 microglia stained with Acridin Orange (scale bar 50 µm) with quantification of green and red (reflecting lysosomal content) fluorescent signal per cell area. (**c**) Representative images of young and aged BV-2 microglia stained with MitoTracker to asses mitochondrial mass. Hoechst 33342 dye was used for nuclear counterstaining (scale bar 50 µm). (**d**) Fluorometric analysis of mitochondrial transmembrane membrane potential (Δym) using TMRE staining. (**e**) Superoxide production in hydrogen peroxide stimulated and unstimulated aged and control BV-2 microglia. Data are mean ± SEM from 5 replicates (**b** to **e**) or 3 replicates (**a**). Statistical annotations $**p < 0.01$ for the indicated comparison.

# Supplementary Figure 4

**a**

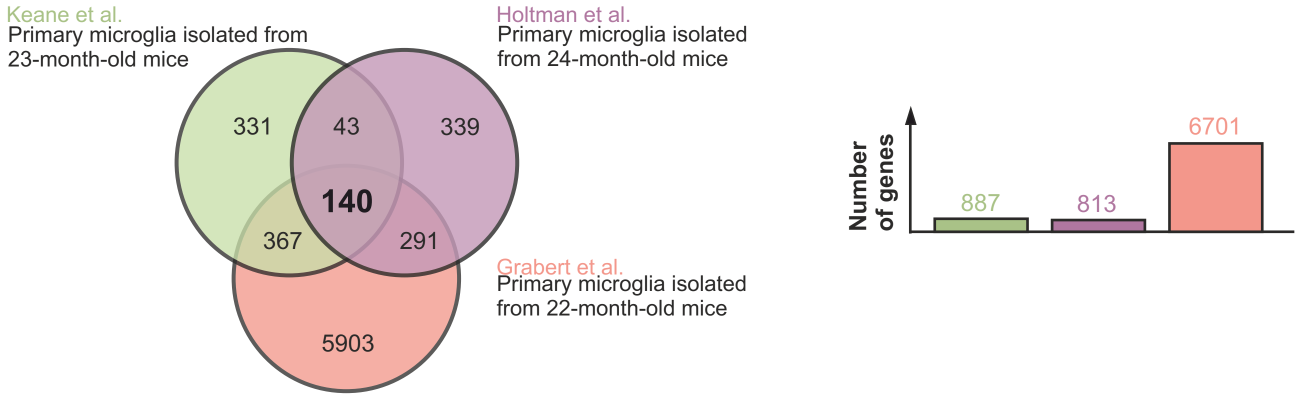


**b**

Overlap between aged BV-2 microglia dataset (1681 genes) and AgingAtlas mouse age-related genes (466 genes)

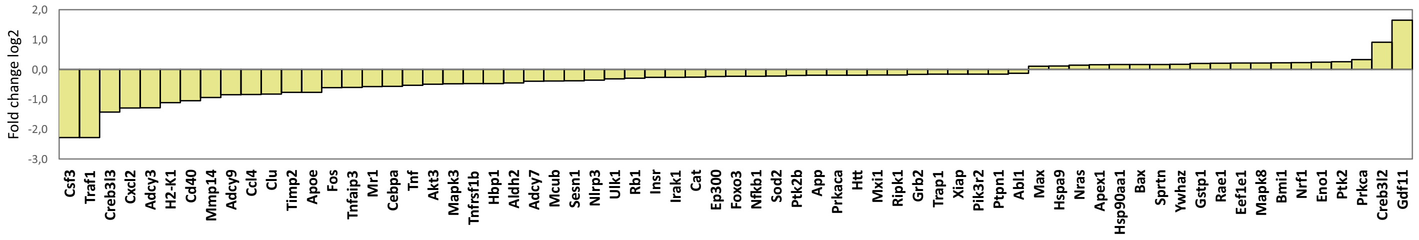


**c**

Overlap between 3 microglia datasets from aged **mouse** (140 genes in total) and AgingAtlas mouse age-related genes (466 genes)

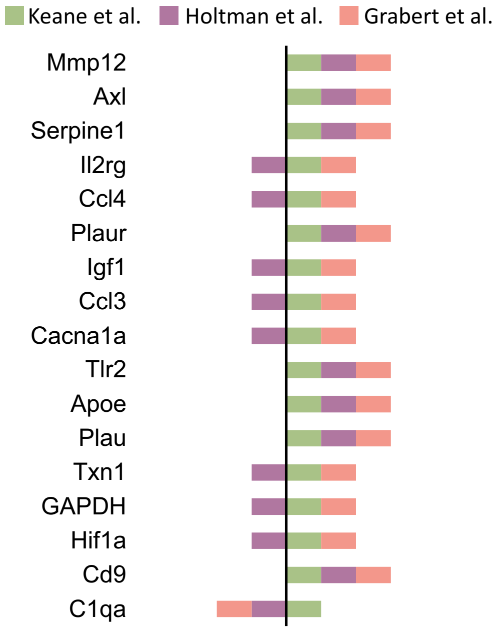


**d**

Overlap between microglia dataset from aged **human** (3769 genes) and AgingAtlas human age-related genes (500 genes)

| | | | | |
|---|---|---|---|---|
| **DOWN** (88 genes) | **CCL4** | APP | TIMP2 | ATF2 |
| | **CCL3** | NFE2L2 | TFDP1 | TERF2 |
| | FLT1 | HOXC4 | TOP2B | TAF1 |
| | PTGS2 | GCLM | **HIF1A** | TNFAIP3 |
| | IL1B | KRAS | RIPK1 | NFKBIA |
| | EGR1 | MED1 | TAB2 | PDGFB |
| | NFKB1 | PRKCA | FOXO3 | XRCC5 |
| | JUND | RPS6KA5 | IL6ST | PRKCB |
| | BCL2A1 | ADIPOR2 | JUN | BMI1 |
| | SESN3 | GSK3B | MAP3K5 | RHEB |
| | NRAS | HSPD1 | PPARG | MAP3K7 |
| | PIK3CA | TOPORS | POLA1 | HBP1 |
| | SIRT1 | PTPN11 | EIF4E | PRKAB2 |
| | EP300 | MAPK9 | ATF4 | HSP90AA1 |
| | FOXO1 | CLOCK | GSR | HSPA9 |
| | BCL2 | BCL10 | SP1 | TPP2 |
| | CDKN1A | WRN | SOD2 | HSPA8 |
| | **PLAU** | TXN | TOP1 | RB1CC1 |
| | AKT3 | GSTA4 | YWHAZ | CTNNB1 |
| | PEX5 | TRAF6 | IRS2 | CCL1 |
| | TLR4 | PTPN1 | NLRP3 | NFKB2 |
| | CEBPB | **PLAUR** | PIK3R1 | A2M |
| **UP** (23 genes) | PIK3R2 | RFC4 | PIN1 | ATF6B |
| | HTRA2 | BAX | MIF | GRN |
| | FOXO4 | RPS6KB2 | GSTP1 | CD14 |
| | TRIM25 | DGAT1 | TSC2 | **C1QA** |
| | JAK2 | GPX4 | HSPA1B | **APOE** |
| | NCOR2 | TOP3B | EIF4EBP1 | |

Cells marked grey share common age-related genes between 3 primary aged mouse datasets and aged human dataset

**Supplementary Figure 4 | Microglial ageing is not connected to classical age-related markers on the transcriptomic level.** (**a**) Venn diagram representing mutual overlap of DEGs between the transcriptome of the three analyzed primary microglia aging mouse studies (Keane et al, 2021; Grabert et al, 2016; Holtmn et al, 2015). (**b**) Comparative analysis of *in vitro* aged BV-2; (**c**) aged mouse microglial datsets (**d**) and human dataset (Olah et al, 2018) together with age-related gene list from AgeingAtlas (Liu et al, 2021).

# Supplementary Figure 5

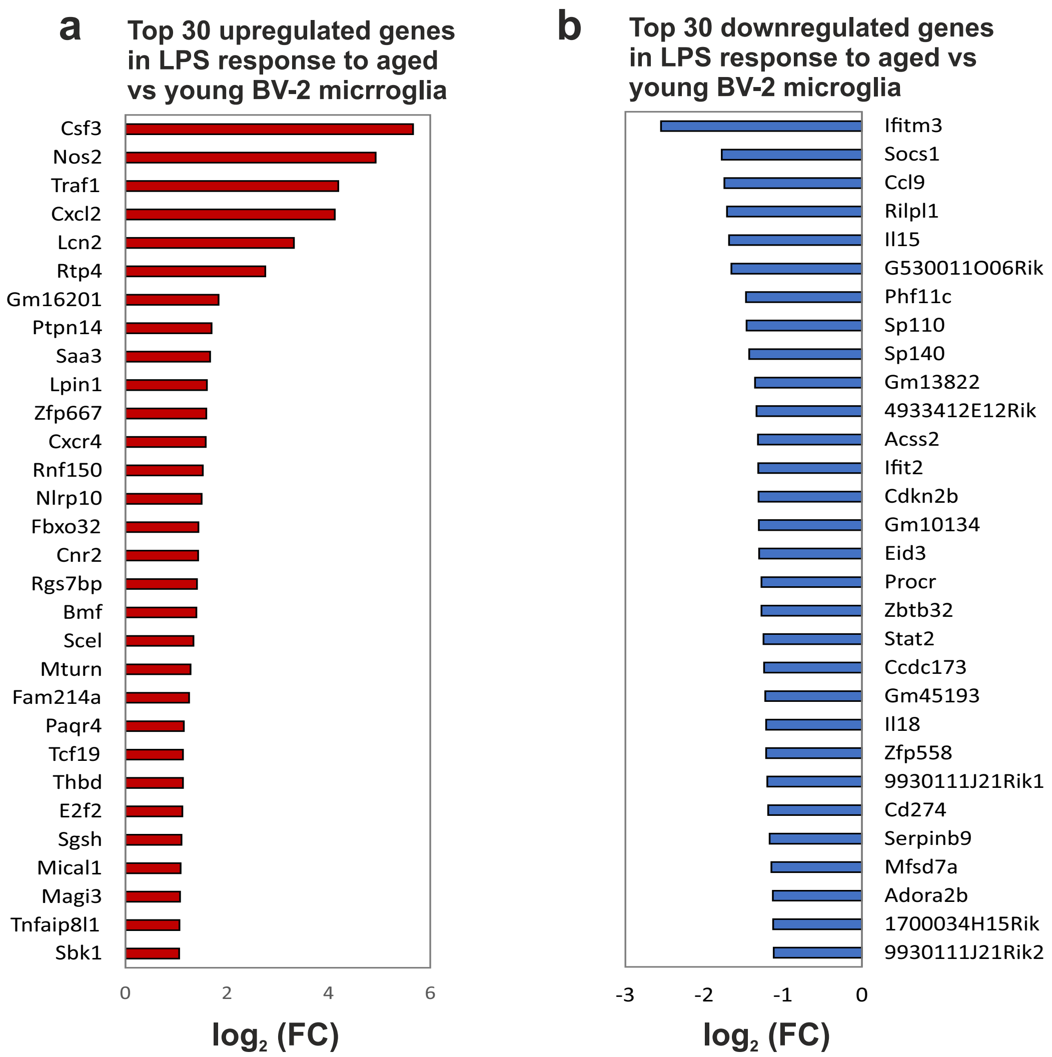


**Supplementary Figure 5** | Graphs display the top 30 upregulated (a) and top 30 downregulated (b) genes in response to LPS treatment in aged BV-2 microglia compared to control BV-2 microglia.

# Supplementary Figure 6

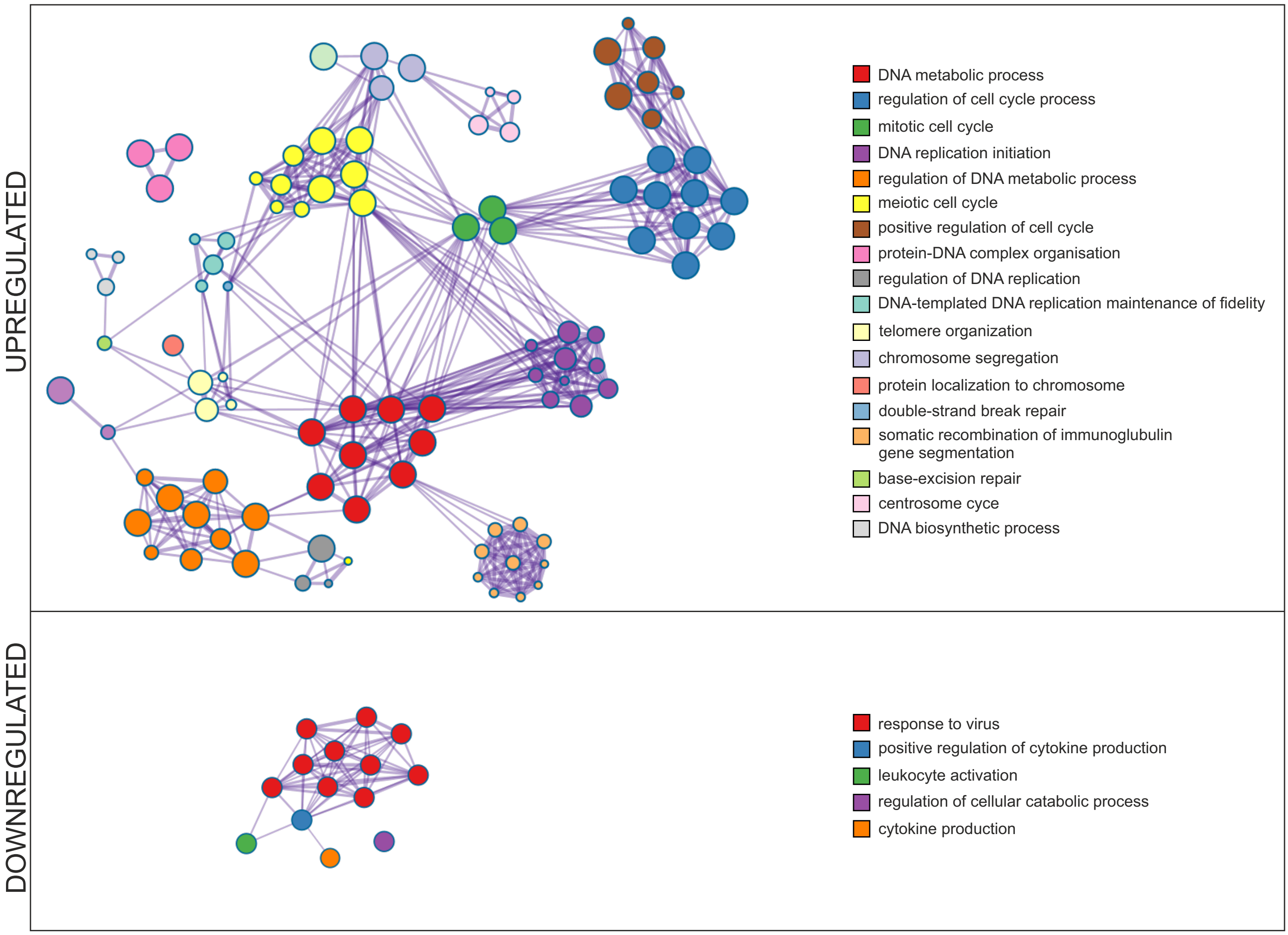


**Supplementary Figure 6** | Cytoscape network analysis of GO terms (with FDR < 0.0001) employing an enrichment map for DEGs in response to LPS treatment between aged BV-2 microglia and control BV-2 microglia. Nodes are grouped by color in clusters based on their similarity. Lines between nodes represent number of genes overlapping in different nodes.

# Supplementary Figure 7

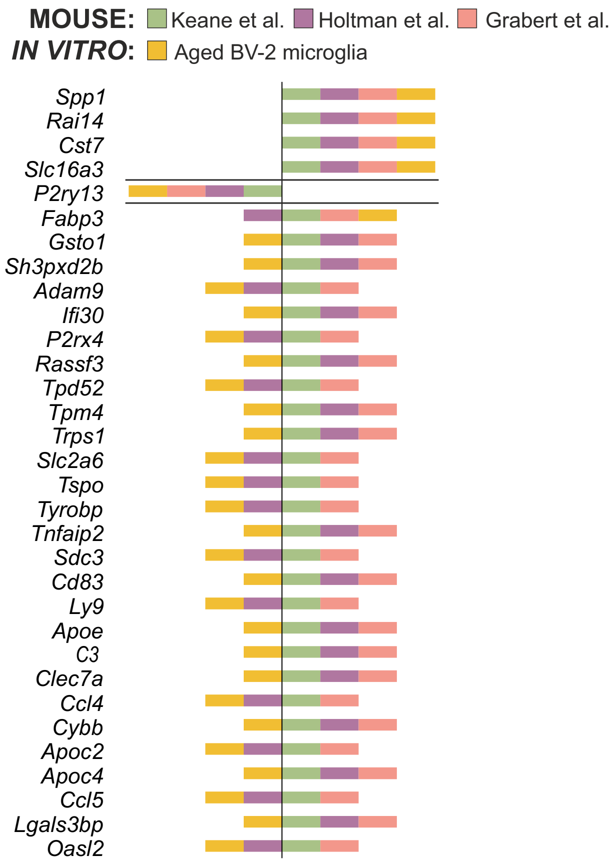


**Supplementary Figure 7** | Graphical representation of the overlap for DEGs between the gene expression profiles obtained from the three primary microglia mouse aging datasets and from the unstimulated aged BV-2 microglia dataset.

# Supplementary Figure 8

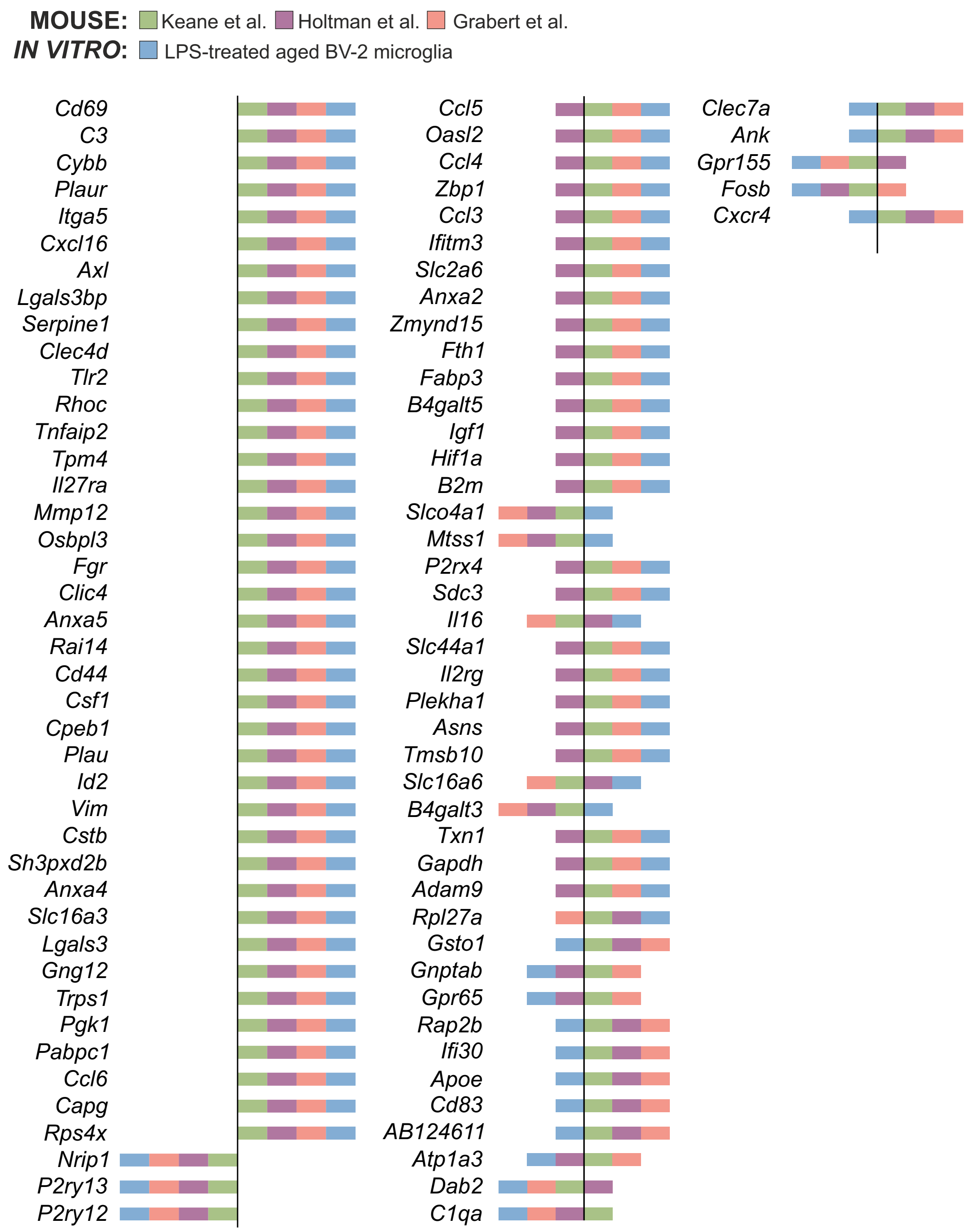


**Supplementary Figure 8** | Graphical representation of the overlap for DEGs between the gene expression profiles obtained from the three primary microglia mouse aging datasets and from the LPS-stimulated aged BV-2 microglia dataset.

# Supplementary Figure 9

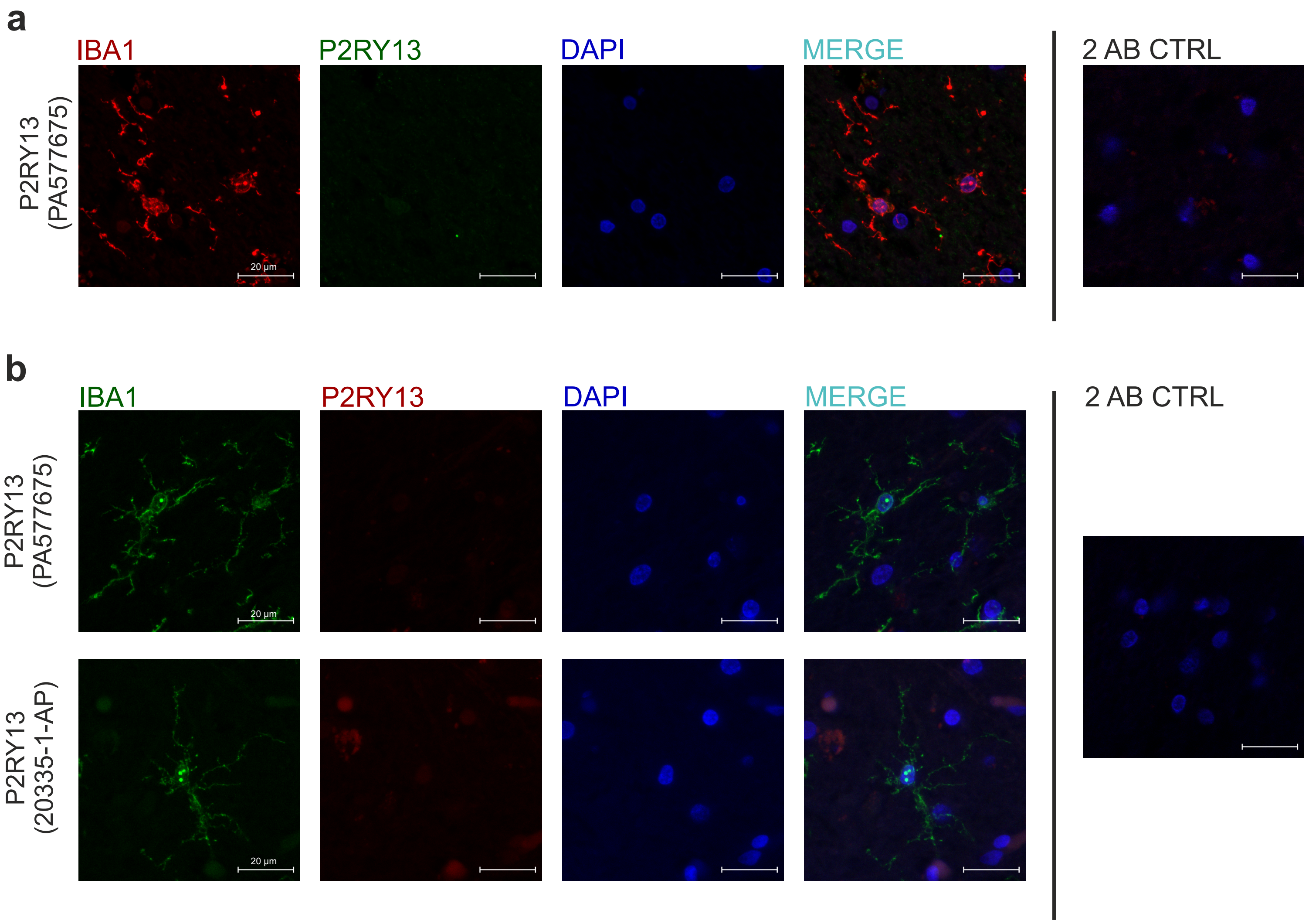


**Supplementary Figure 9** |Commercially available antibodies directed against human P2RY13 protein (**a**), including with the use of a booster to enhance the immunofluorescence signal (**b**) failed to yield satisfactory quality and to generate a positive fluorescent signal in our immunofluorescent analyses in human brain tissues. Scale bar 20 µm.

# Supplementary Figure 10

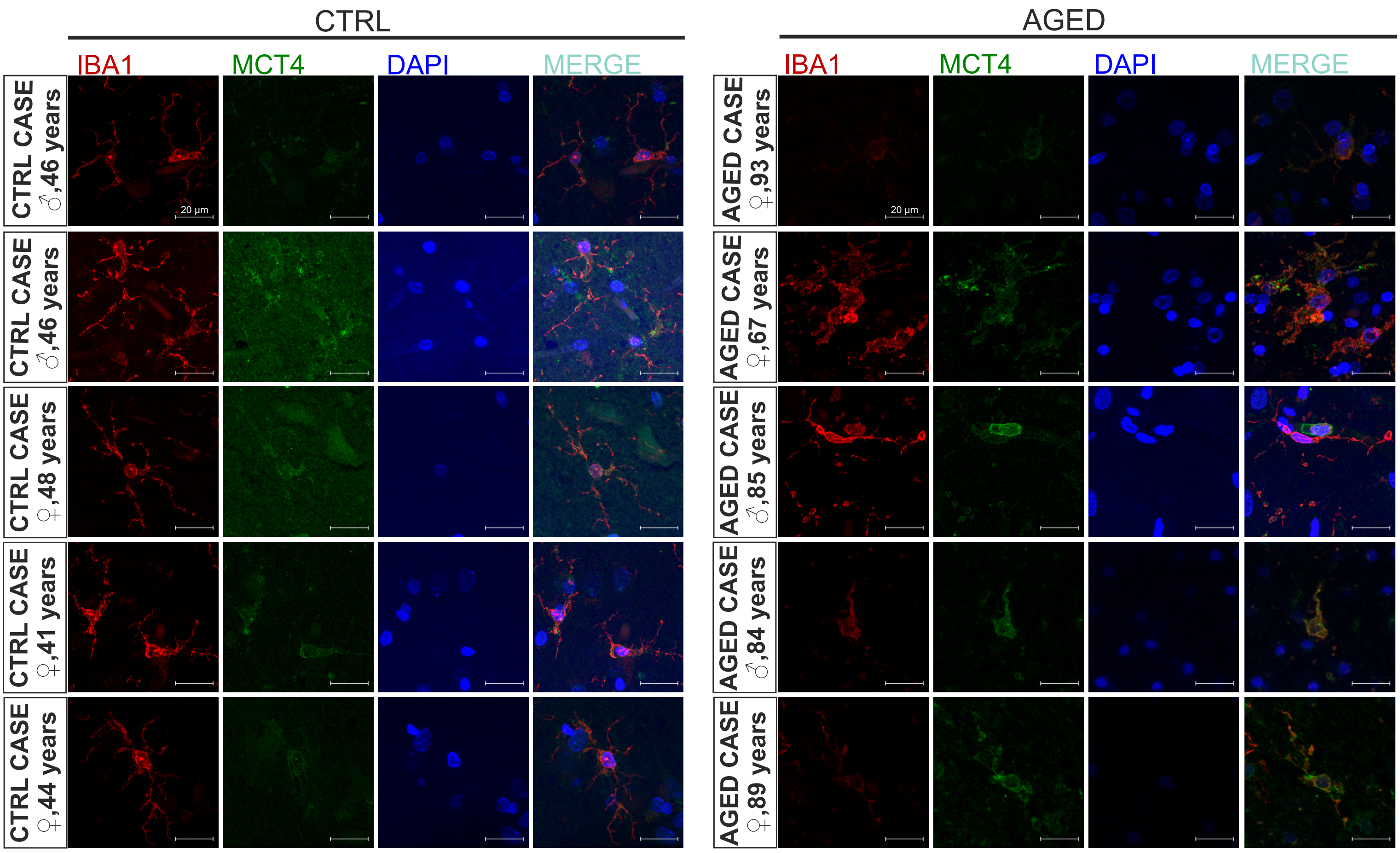


**Supplementary Figure 10** | Complement to Figure 7a to d, immunofluorescence analysis of microglial MCT4 expression in human brain tissues - 5 cases per age group are depicted. Scale bar 20 µm.

# Supplementary Figure 11

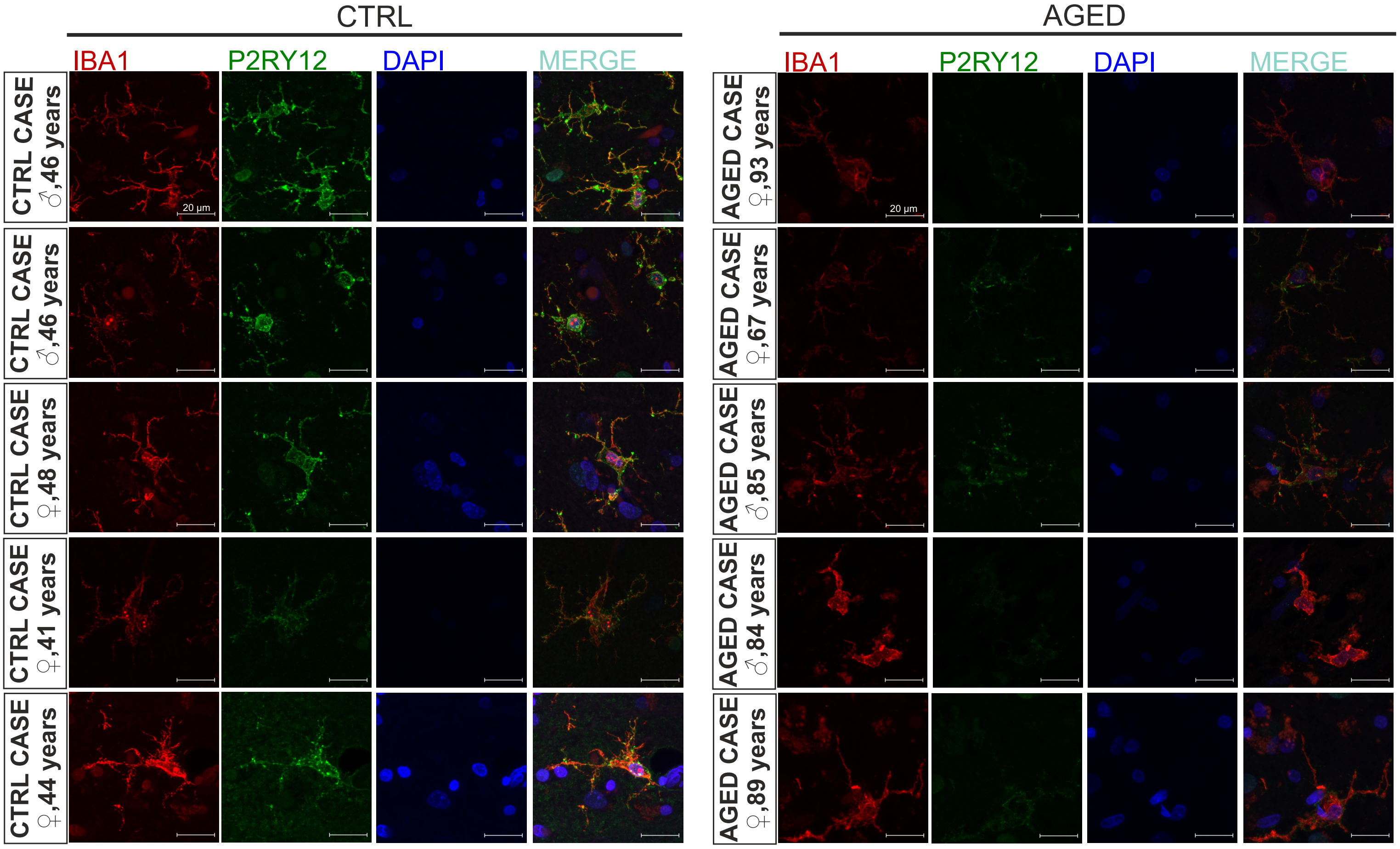


**Supplementary Figure 11** | Complement to Figure 7e to h, immunofluorescence analysis of microglial P2RY12 expression in human brain tissues - 5 cases per age group are depicted. Scale bar 20 µm.

# Supplementary Figure 13

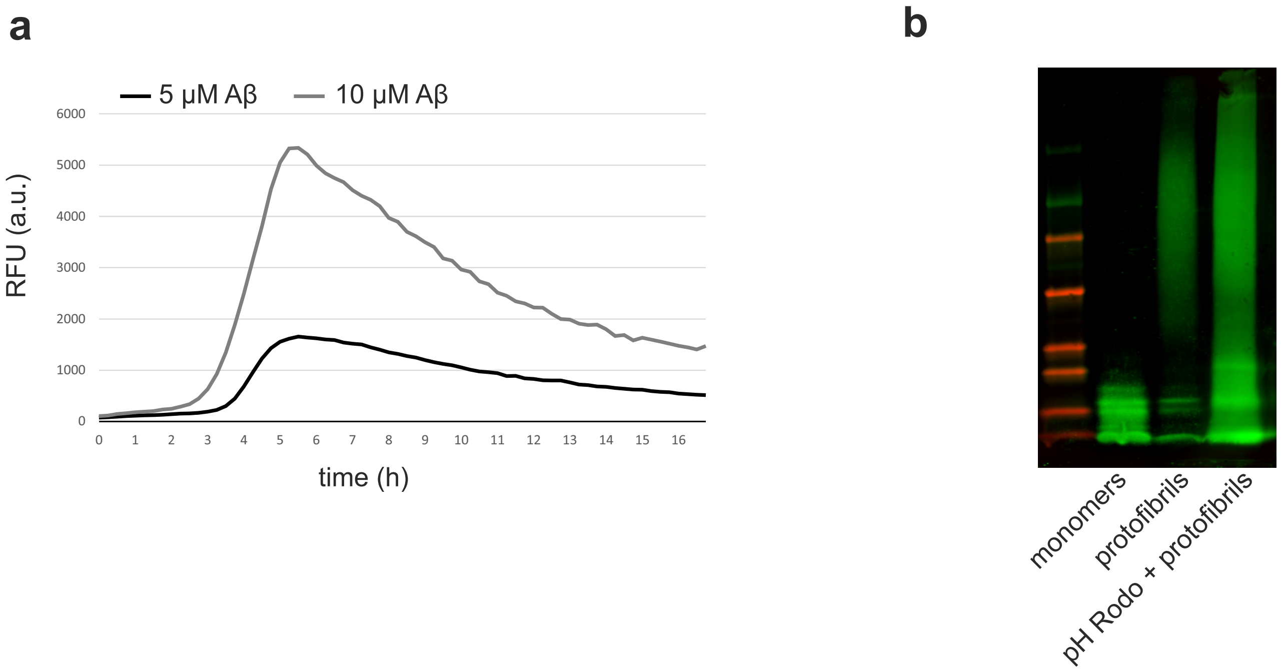


**Supplementary Figure 13** | (**a**) Thioflavin assay showing aggregation of Amyloid-β over time. (**b**) Immunoblot analysis of Amyloid-β monomers, unstained protofibrils and pHrodo Red stained protofibrils.

# Supplementary Figure 12

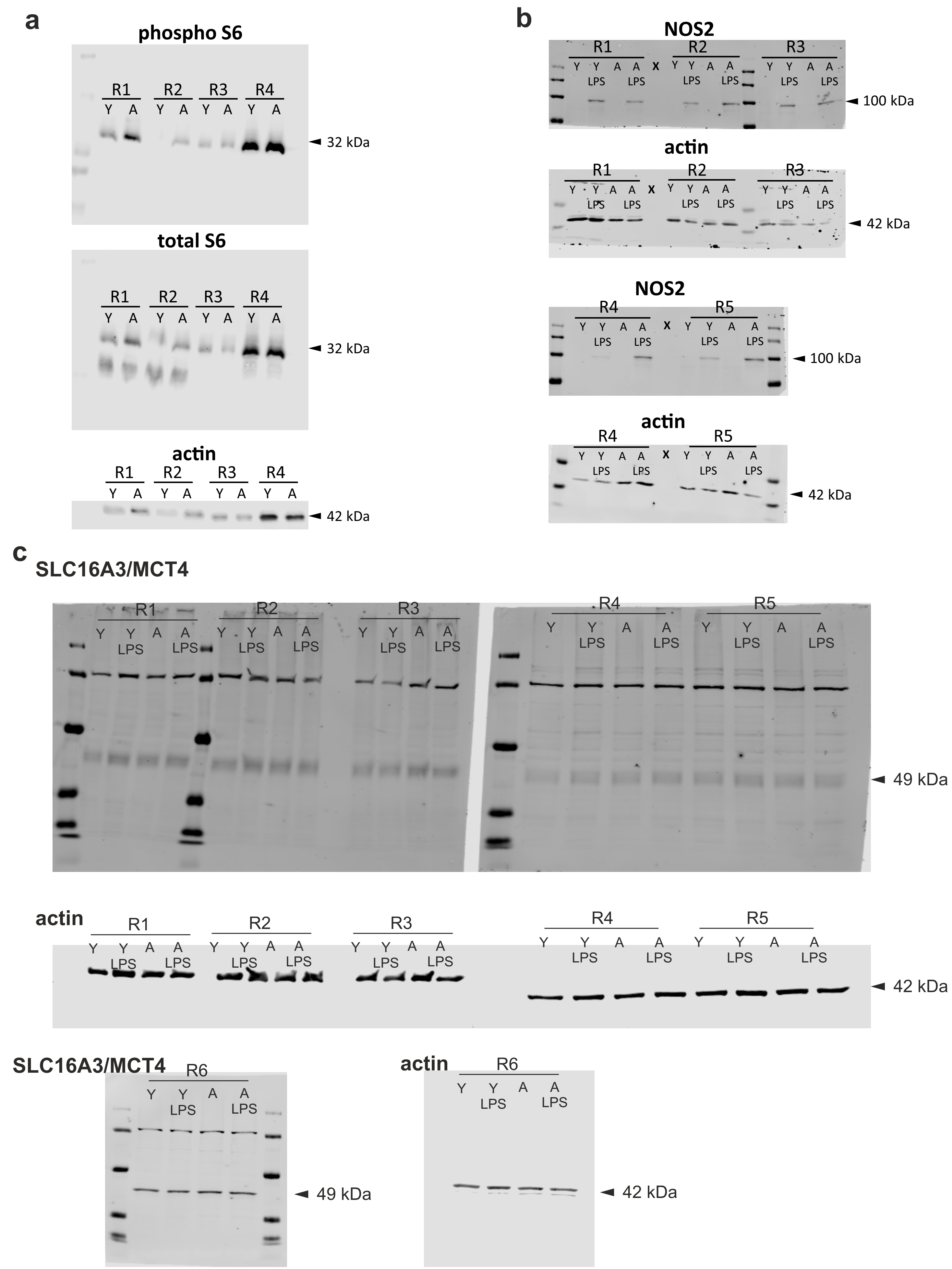


**Supplementary Figure 12** | Full uncropped Immunoblot membranes for all replicates.

# Supplementary Figure 14

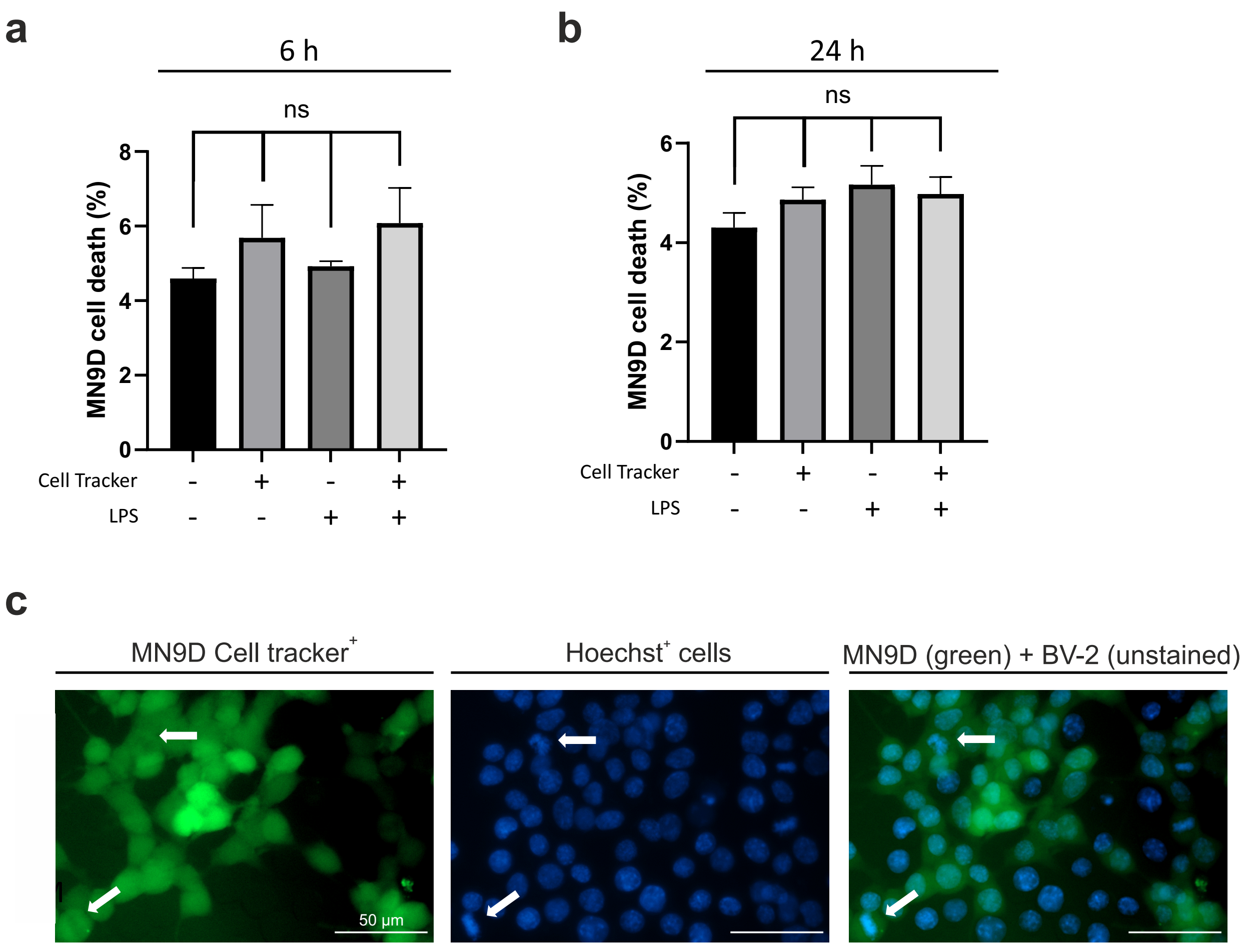


**Supplementary Figure 14** | The MN9D neural monoculture subjected to exposure of experimental conditions of CellTracker or LPS alone, or both together, examined for neurotoxicity after 6 hours (**a**) or 24 hour time point (**b**). Microscopic images depicting damaged MN9D neural nuclei, highlighted by white arrows, observed in co-culture with BV-2 microglia (**c**). Scale bar = 50 µm.
