# Supplementary material for: Age-associated microglial transcriptome leads to diminished immunogenicity and dysregulation of MCT4 and P2RY12/P2RY13 related functions
Source: Cell Death Discov. 2025 Jan 19;11:16. doi: 10.1038/s41420-025-02295-1 (PMC11743796; doi:10.1038/s41420-025-02295-1)
Supplement: Supplementary file 2 — Original data [file 41420_2025_2295_MOESM2_ESM.pdf]

Supplementary Figure 12

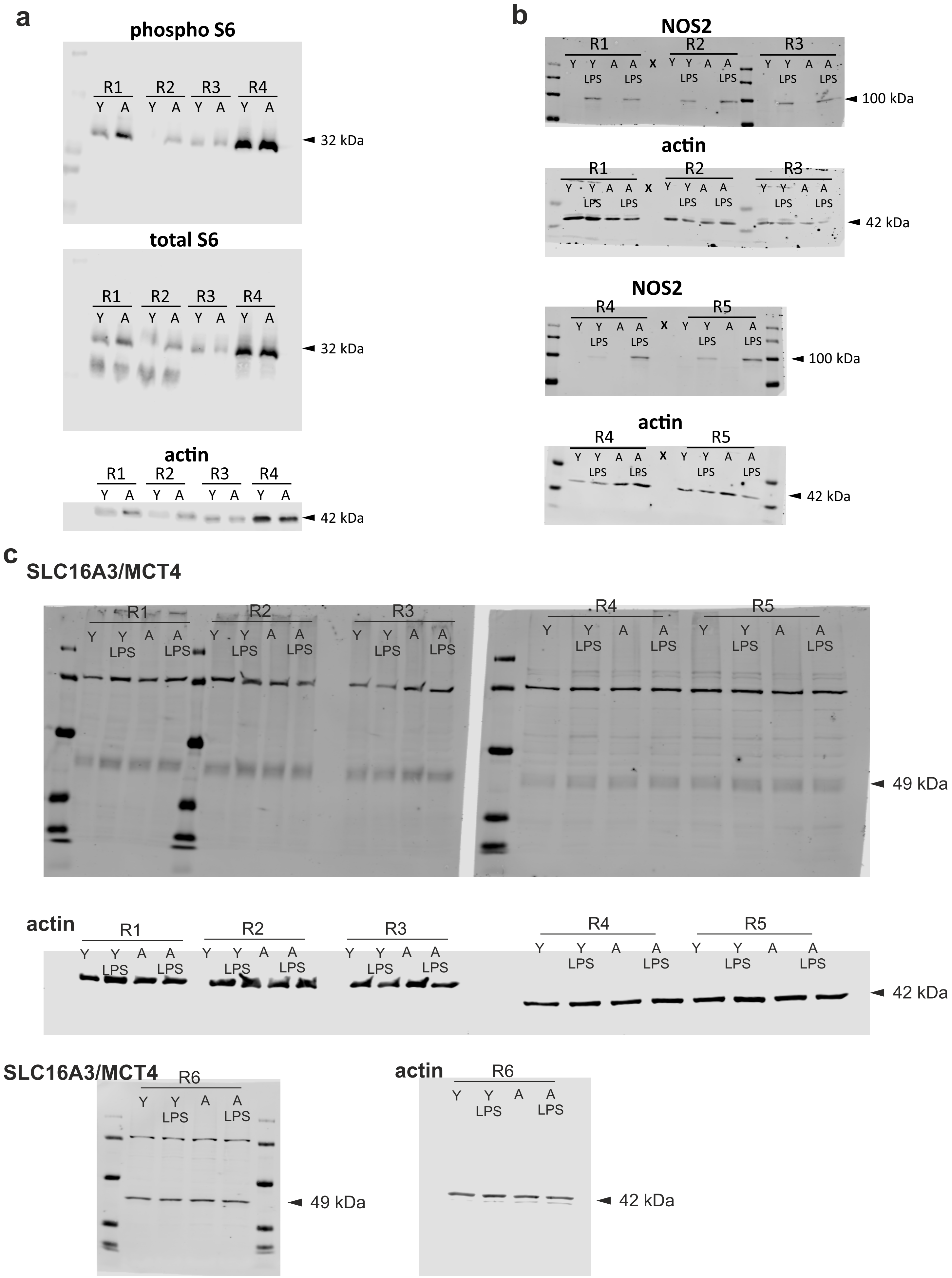

Supplementary Figure 12 | Full uncropped Immunoblot membranes for all replicates.
